# In vivo evaluation of guide-free Cas9-induced safety risks in a pig model

**DOI:** 10.1038/s41392-024-01905-1

**Published:** 2024-07-19

**Authors:** Weikai Ge, Shixue Gou, Xiaozhu Zhao, Qin Jin, Zhenpeng Zhuang, Yu Zhao, Yanhui Liang, Zhen Ouyang, Xiaoyi Liu, Fangbing Chen, Hui Shi, Haizhao Yan, Han Wu, Liangxue Lai, Kepin Wang

**Affiliations:** 1 China–New Zealand Joint Laboratory on Biomedicine and Health, CAS Key Laboratory of Regenerative Biology, Guangdong Provincial Key Laboratory of Stem Cell and Regenerative Medicine, Joint School of Life Sciences, Guangzhou Institutes of Biomedicine and Health, Chinese Academy of Sciences, Guangzhou Medical University, Guangzhou, 510530 China; 2 Sanya institute of Swine resource, Hainan Provincial Research Center of Laboratory Animals, Sanya, 572000 China; 3 Guangdong Provincial Key Laboratory of Large Animal models for Biomedicine, Wuyi University, Jiangmen, 529020 China; 4 Guangzhou National Laboratory, Guangzhou, 510005 China; 5 University of Chinese Academy of Sciences, 100049 Beijing, China; 6 Research Unit of Generation of Large Animal Disease Models, Chinese Academy of Medical Sciences (2019RU015), Guangzhou, 510530 China

**Keywords:** Gene therapy, Genomic instability

## Abstract

The CRISPR/Cas9 system has shown great potential for treating human genetic diseases through gene therapy. However, there are concerns about the safety of this system, specifically related to the use of guide-free Cas9. Previous studies have shown that guide-free Cas9 can induce genomic instability in vitro. However, the in vivo safety risks associated with guide-free Cas9 have not been evaluated, which is necessary for the development of gene therapy in clinical settings. In this study, we used doxycycline-inducible Cas9-expressing pigs to evaluate the safety risks of guide-free Cas9 in vivo. Our findings demonstrated that expression of guide-free Cas9 could induce genomic damages and transcriptome changes in vivo. The severity of the genomic damages and transcriptome changes were correlate with the expression levels of Cas9 protein. Moreover, prolonged expression of Cas9 in pigs led to abnormal phenotypes, including a significant decrease in body weight, which may be attributable to genomic damage-induced nutritional absorption and metabolic dysfunction. Furthermore, we observed an increase in whole-genome and tumor driver gene mutations in pigs with long-term Cas9 expression, raising the risk of tumor occurrence. Our in vivo evaluation of guide-free Cas9 in pigs highlights the necessity of considering and monitoring the detrimental effects of Cas9 alone as genome editing via the CRISPR/Cas9 system is implemented in clinical gene therapy. This research emphasizes the importance of further study and implementation of safety measures to ensure the successful and safe application of the CRISPR/Cas9 system in clinical practice.

## Introduction

The CRISPR/Cas9 system, consisting of the Cas9 protein guided by single guide RNAs (sgRNAs), has emerged as the leading genome editing tool in various organisms.^1,2^ Cas9 can precisely target specific genomic sequences and efficiently induce DNA double-stranded breaks (DSBs). These DSBs can be repaired through nonhomologous end joining (NHEJ) or template-dependent homology-directed repair, resulting in the generation of random insertions or deletions (indels) or precise point mutations and exogenous DNA fragment insertions, respectively. The CRISPR/Cas9 system has been extensively employed in generating animal models of human diseases, studying genotype-phenotype relationships, and improving crop and livestock traits.^3–5^ Moreover, CRISPR/Cas9-mediated gene therapy has been applied in clinical trials for treating various genetic diseases or refractory conditions,^6^ such as sickle cell disease,^7^ β-thalassemia,^7,8^ transthyretin amyloidosis,^9^ retinal degeneration,^10^ acquired immunodeficiency syndrome,^11^ and cancers.^11,12^ However, despite promising clinical proof of concept, certain clinical trials have faced delays or cancellations due to significant safety concerns, including off-target risks,^13^ unexpected chromosomal rearrangements and chromothripsis,^14–20^ activation of p53-mediated DNA damage response,^21–23^ and potential immunogenicity.^24–27^

While concerns regarding sgRNA-dependent “off-target” DSBs have garnered significant attention, strategies such as the use of sgRNAs with low off-target activity, high fidelity Cas9 variants, or alternative gene editing approaches, such as double-nickase, can reduce this outcome.^28^ However, it has been reported that Cas9 protein alone can function as a genome mutator independent of exogenous sgRNA, leading to genomic DNA DSB damages in human cells.^29^ Xu et al. demonstrated the underlying mechanism by which Cas9 interacts with the KU86 subunit of the DNA-dependent protein kinase (DNA-PK) complex, disrupting the interaction between KU86 and its kinase subunit. This disruption impairs DNA-PK-dependent repair of DNA DSB damage through the NHEJ pathway. Another study revealed that Cas9 expression in cell lines can activate the p53 pathway and promote the accumulation of p53-inactivating mutations.^30^ In our previous report, we found that Cas9 protein induced genome damages and transcriptome changes in pig embryos in vitro, which could impair preimplantation embryonic development.^31^ These findings raise additional safety concerns regarding the use of the CRISPR/Cas9 system in clinical applications. However, thus far, the evaluation of guide-free Cas9-mediated safety concerns has been limited to in vitro studies. The assessment of whether the expression of exogenous Cas9 protein alone can induce genomic instability and activate the DNA damage response (DDR) pathway in vivo is critical, particularly for in vivo gene therapy. Such clarification in animal models is necessary.

Pigs and humans share similar physiology and immune systems,^32,33^ making pigs suitable animal models for studying the safety and efficacy of gene therapy.^34,35^ Previously, we established a controllable and switchable transgene expression system in pigs,^36^ and generated a pig model with doxycycline (Dox)-inducible Cas9 expression.^37^ This Dox-inducible Cas9-expressing (DIC) pig model offers unique advantages for this study. First, this system only expresses Cas9 protein after Dox administration, eliminating the interference effects of sgRNAs. Second, the expression of the Cas9 protein in vivo does not require delivery cargos such as lentivirus, adeno-associated viruses, or lipid nanoparticles, thereby increasing efficiency and excluding the interference effects of delivery cargos. Last, the expression level and duration of Cas9 protein can be tightly regulated by Dox administration, enabling evaluation of the correlation between negative effects and the expression time and level of Cas9 protein. Therefore, the DIC pig line represents an ideal large animal model for investigating the detrimental effects of guide-free Cas9 in vivo.

Our findings indicate that significant genomic damages and transcriptome changes occurred in blood cells and tissues after two weeks of Cas9 expression. Moreover, continuous inducible Cas9 expression resulted in significantly lower body weights in DIC pigs compared to wild-type (WT) pigs, potentially due to abnormal stomach and intestinal functions induced by long-term Cas9 expression. Whole-genome sequencing (WGS) analysis of these DIC pigs with abnormal phenotypes revealed an increased number of genome mutations across the entire genome (including 568 cancer driver genes) compared to that in WT pigs, suggesting an increased risk of tumorigenesis. Overall, these in vivo pig data emphasize the need to consider the safety of the Cas9 protein alone as CRISPR/Cas9-mediated gene therapy progresses towards clinical applications.

## Results

### Expression of Cas9 in DIC pigs induces random DSBs in the genome

To realize the autonomous and interference-free expression of Cas9 in vivo, we previously generated a Dox-inducible Cas9 endogenous expression transgenic pig line using somatic cell nuclear transfer.^37^ The rtTA (Tet-on 3G) expression cassette and TRE3G-Cas9-T2A-tdTomato expression cassette were inserted into the porcine *Rosa26* and *Hipp11* loci, respectively (Supplementary Fig. 1a). Cas9 expression could be flexibly regulated by Dox administration, and tdTomato served as a reporter gene downstream of Cas9 (Supplementary Fig. 1b). We first treated DIC pigs and age-matched WT pigs with oral administration combined with intraperitoneal injection of Dox for two weeks and collected blood samples at various time points (day 0, day 3, day 9, and day 14). Flow cytometry analysis showed that 0.093%, 16.6%, 36.8%, and 41.5% of peripheral blood mononuclear cells (PBMCs) in DIC pigs expressed tdTomato after Dox administration of day 0, day 3, day 9, and day 14, respectively (Supplementary Fig. 1c), with tdTomato-positive PBMCs co-expressing the Cas9 protein (Supplementary Fig. 1b). No expression of tdTomato was observed in the PBMCs of DIC pigs without Dox administration and WT pigs with/without Dox administration (Supplementary Fig. 1c). These results indicate that Cas9 expression in DIC pigs can be tightly controlled by Dox administration and visualized through red fluorescence.

**Fig. 1:**
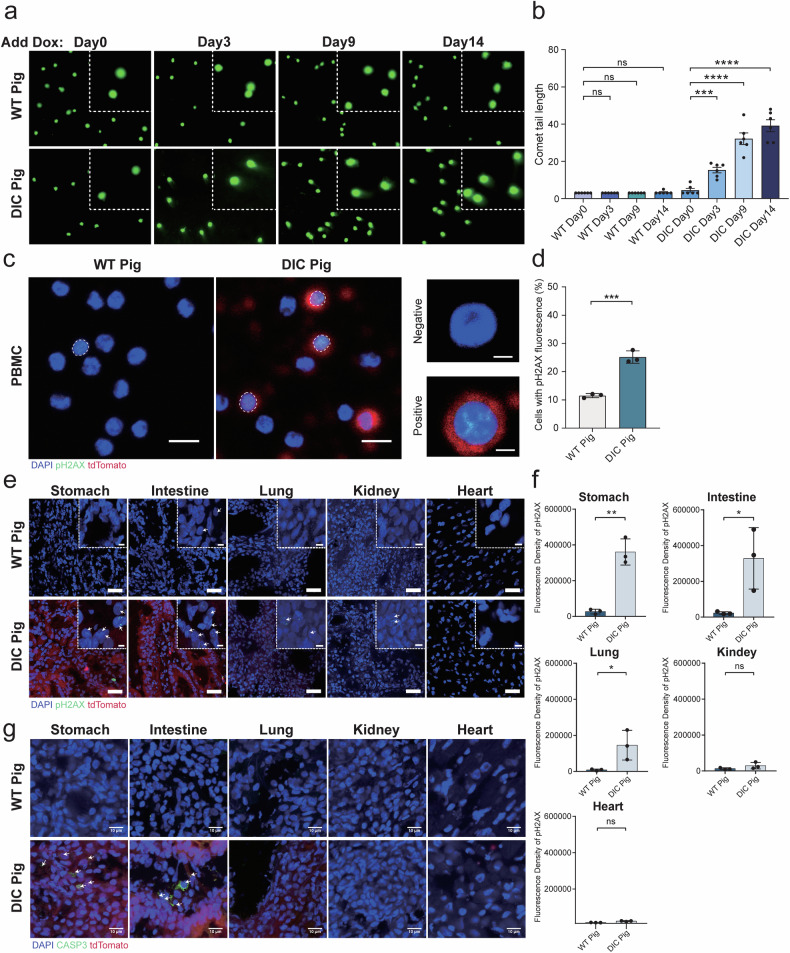
Analysis of guide-free Cas9-induced genomic damages in pig model. **a** Comet assay detection of the PBMCs of WT and DIC pigs on Day 0, Day 3, Day 9 and Day 14 of Dox administration. **b** Quantification of the tail length of the PBMCs of the WT and DIC pigs at four time points. ***p = 0.0001, ****p < 0.0001, *n* = 6, unpaired *t* test. **c** Fluorescence microscopy images of pH2AX (green), tdTomato (red) and DAPI (blue) in WT and DIC pig PBMCs. Genome damaged cells are marked by the white dashed circle, scale bar = 10 μm. Negative indicates a normal cell, and positive indicates a cell with DNA DSBs, scale bar = 2 μm. **d** Percentage of pH2AX-positive cells in the PBMCs of WT pigs and DIC pigs, ***p = 0.0005, *n* = 3, unpaired *t* test. **e** Fluorescence microscope images of pH2AX (green), tdTomato (red), and DAPI (blue) in the stomach, intestine, lungs, kidneys, and heart of the WT and DIC pigs; white arrows indicate genome-damaged cells, scale bar = 20 μm, area bar = 5 μm. **f** Quantification of the fluorescence density of pH2AX in the stomach, intestine, lungs, kidneys, and heart of the WT and DIC pigs from three independent repeats. *p < 0.0332, **p < 0.0021, *n* = 3, unpaired *t* test. Scale bar = 10 μm. **g** Fluorescence microscope images of CASP3 (green), Cas9 (red), and DAPI (blue) in the stomach, intestine, lungs, kidneys, and heart of the WT and DIC pigs; white arrows indicate apoptotic cells. Data in **b**, **d**, and **f** are presented as mean ± SEMs

Next, we selected three DIC pigs and three age-matched WT pigs to investigate whether the Cas9 protein induces genomic damages in vivo. We used a nuclear comet assay to analyze genomic damages in the PBMCs of DIC and WT pigs. Nuclear comet tails were observed in the PBMCs of DIC pigs on Day 3, Day 9, and Day 14 after Dox administration but not on Day 0 or in WT pigs (Fig. 1a). Statistical analysis of the average tail lengths and comet cell rates, which indicate the severity and extent of genomic damages, respectively, revealed that genomic damages in the PBMCs of DIC pigs were gradually increased with Dox administration and PBMCs with Dox treatment of 14 days exhibited the longest tail length and highest number of cells with comet tails (average tail lengths, Day 0 : Day 3 : Day 9 : Day 14 = 4.5 ± 1.0 : 15.3 ± 1.5: 32.2 ± 3.1 : 39.2 ± 3.2; comet cell rates, Day 0 : Day 3 : Day 9 : Day 14 = 1.3% ± 0.7% : 28.1% ± 5.2% : 51.3% ± 6.3% : 61.7% ± 4.8%) (Fig. 1b, Supplementary Fig. 2a). However, no significance difference of average tail lengths and comet cell rates was observed in the PBMCs of WT pigs before and after Dox administration (average tail lengths, Day 0 : Day 3 : Day 9 : Day 14 = 3.0 ± 0.0 : 3.0 ± 0.0 : 3.0 ± 0.0 : 3.3 ± 0.3; comet cell rates, Day 0 : Day 3 : Day 9 : Day 14 = 1.8% ± 0.9% : 1.7% ± 0.8% : 1.9% ± 1.1% : 2.9% ± 0.8%).Then, we analyzed the formation of phosphorylated H2AX (pH2AX), a marker for DNA DSBs, in the PBMCs of DIC pigs on day 14 after Dox administration. The pH2AX signal highly co-localized with the tdTomato signal, indicating the presence of the Cas9 protein (Fig. 1c). The number of PBMCs with DNA DSBs was significantly higher in DIC pigs (25.2% ± 2.2%) than in WT pigs (11.5% ± 0.7%), as determined by counting pH2AX-positive cells (Fig. 1d). These findings suggested that Cas9 induces DNA DSBs in the PBMCs of DIC pigs in vivo, with the severity of the genomic damages correlating with Cas9 expression time and the number of cells with genomic damages correlating with the number of cells expressing Cas9 protein.

To determine whether the Cas9 protein can cause genomic damages in solid organs, we euthanized three DIC pigs and three age-matched WT pigs treated with Dox for 2 weeks and collected tissues from the stomach, intestine, lungs, kidneys, and heart. Given that the accessibility and inducibility of the Dox-inducible system in different organs might vary, we assessed the expression levels of Cas9 in these organs. Quantitative PCR and immunohistochemical (IHC) staining results confirmed that high Cas9 expression in the stomach and intestine, moderate Cas9 expression in the lungs and kidneys, and low Cas9 expression in the heart (Supplementary Fig. 2b, c). Consistent with the Cas9 expression levels, we discerned pH2AX foci in the stomach, intestine, lungs, and kidneys using fluorescence microscopy, but infrequent in the heart (Fig. 1e). The fluorescence density of pH2AX indicated significant genomic damages in the stomach (WT Pig : DIC Pig = 27,065 ± 13,299 : 360,484 ± 72,904), intestine (WT Pig : DIC Pig = 22,712 ± 7,309 : 328,639 ± 171,570), and lungs (WT Pig : DIC Pig = 8,346 ± 4,607 : 145,666 ± 82,312), whereas genomic damages in the kidneys (29,351 ± 10,406) and heart (22,492 ± 1,426) of DIC pigs were comparable to WT pigs (kidneys: 13,051 ± 2,658; heart: 14,492 ± 283) (Fig. 1f). Furthermore, immunofluorescence (IF) staining for CASP3, an apoptotic marker, revealed the presence of apoptotic cells in the stomach and intestine of DIC pigs treated with Dox but rare in the lungs, kidneys, or heart (Fig. 1g). These results demonstrated that Cas9 protein could induce DNA DSBs in solid organs in vivo, with a positive correlation between genomic damages and Cas9 expression levels.

To further exclude the potential impact of the tdTomato protein present in DIC pigs, the previously established Dox-inducible tdTomato-expressing (DITD) porcine fetal fibroblasts (PFFs) cell line (Supplementary Fig. 3a),^36^ in which the rtTA expression cassette and TRE3G-tdTomato expression cassette were inserted into the porcine *Rosa26* and *Hipp11* loci, respectively, was used as a control to verify Cas9-induced genomic damages in vitro. DIC, DITD, and WT PFFs were cultured in the medium supplemented with/without Dox. After 3 days of Dox treatment, 40.8% of the DITD PFFs expressed tdTomato and 19.0% of the DIC PFFs simultaneously expressed tdTomato and Cas9, but the WT PFFs had no detectable tdTomato and Cas9 expression (Supplementary Fig. 3b). We then verified that genomic damages were caused by the expression of Cas9 but not tdTomato via nuclear comet assay in vitro. Nuclear comet tails were only observed in the DIC PFFs, but not in the DITD PFFs or WT PFFs after Dox treatment (Supplementary Fig. 3c). Statistical analysis showed that the average tail lengths and comet cell rates were gradually increased with the extending time of Dox treatment in the DIC PFFs (average tail lengths, 0 h : 24 h : 48 h : 72 h = 3.0 ± 0.0 : 12.4 ± 2.5 : 16.8 ± 0.9 : 29.0 ± 1.1; comet cell rates, 0 h : 24 h : 48 h : 72 h = 0.4% ± 0.4% : 33.1% ± 3.7% : 42.0% ± 4.1% : 79.8% ± 1.8%) (Supplementary Fig. 3d, e). In contrast, the average tail lengths and comet cell rates of the DITD PFFs with Dox treatment (average tail lengths, 0 h : 24 h : 48 h : 72 h = 3.0 ± 0.0 : 3.0 ± 0.0 : 3.0 ± 0.0 : 3.4 ± 0.4; comet cell rates, 0 h : 24 h : 48 h : 72 h = 1.3% ± 0.9% : 2.1% ± 0.7% : 6.7% ± 0.2% : 3.2% ± 1.7%) did not change significantly compared to the WT PFFs (average tail lengths, 0 h : 24 h : 48 h : 72 h = 3.0 ± 0.0 : 3.0 ± 0.0 : 3.0 ± 0.0 : 3.0 ± 0.0; comet cell rates, 0 h : 24 h : 48 h : 72 h = 0.7% ± 0.5% : 2.9% ± 1.5% : 3.7% ± 1.6% : 3.4% ± 1.2%) (Supplementary Fig. 3d, e). IF staining further showed that pH2AX foci were detected and gradually became more prominent with extending Dox treatment time in the DIC PFFs (0 h : 24 h : 48 h : 72 h = 11,505 ± 4,707, 154,207 ± 15,851, 272,168 ± 18,409, 539,035 ± 53,762). Conversely, pH2AX foci were barely detectable in both DITD PFFs (0 h : 24 h : 48 h : 72 h = 6,235 ± 5,262, 12,340 ± 9,353, 55,404 ± 24,339, 27,468 ± 6,228) and WT PFFs (0 h : 24 h : 48 h : 72 h = 386 ± 270, 25,995 ± 13,880, 19,568 ± 11,764, 2,822 ± 2,711) (Supplementary Fig. 3f, g). These results confirmed that the observed genomic damages in DIC pigs were a direct consequence of Cas9 protein expression, not related to the expression of tdTomato.

Previous studies have shown that Cas9-induced DNA DSBs can activate the expression of the *P53* gene, leading to the increased expression of downstream target genes such as *PERP*, *P21*, *PUMA*, and *NOXA* in human cells in vitro.^29,30^ Therefore, we investigated the expression of these *P53* target genes in PBMCs and solid organs in vivo (Supplementary Figs. 4, 5). Consistent with findings from experiments of human cells, we observed the activation of some *P53* downstream target genes in the PBMCs of DIC pigs treated with Dox for 3 and 14 days, as well as in Cas9-expressing tissues (stomach, intestine, and kidneys) (Supplementary Figs. 4a, 5a). Analysis of the summary scores for the expression of these *P53* target genes showed significant differences between DIC pigs and WT pigs treated with Dox in PBMCs on Day 14 and in the stomach, which exhibited the longest Cas9 expression in blood cells and the highest Cas9 expression in tissues (Supplementary Figs. 4b, 5b). These findings further support a relationship between genomic damages and the duration and expression levels of Cas9 in PBMCs and solid organs.

### Transcriptome homeostatic changes in the PBMCs of DIC pigs with Cas9 expression

**Fig. 2:**
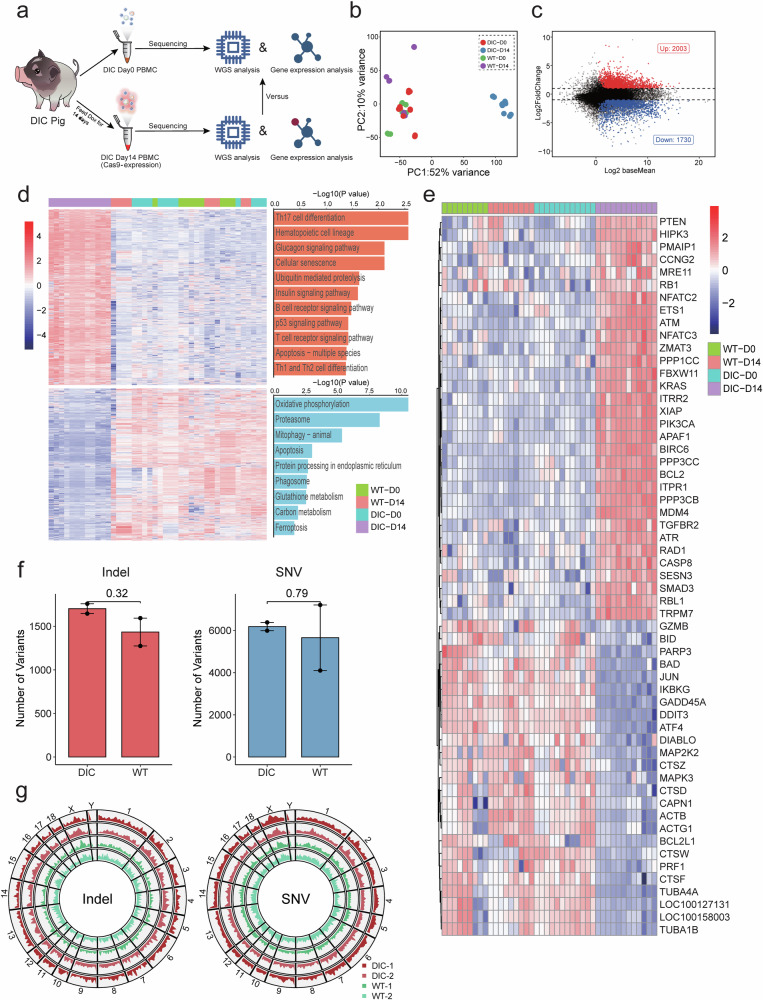
Transcriptome sequencing and whole-genome sequencing of PBMCs from DIC pigs after two weeks of Dox feeding. **a** Schematic diagram showing Cas9 expression induced by Dox followed by transcriptome or genome sequencing in DIC pigs. **b** Principal component analysis of RNA-Seq samples from the PBMCs of the DIC and WT pigs after administration of Dox for 0 (D0) and 14 days (D14). **c** The MA plot shows the up- and downregulated differentially expressed genes in the PBMCs of the DIC pigs after administration of Dox for 14 days. Red points represent the upregulated differentially expressed genes (log2FoldChange > 1 and *p*-adjusted-value < 0.05), blue points represent the downregulated differentially expressed genes (log2FoldChange < 1 and *p*-adjusted-value < 0.05), and black points indicate genes with no significant difference in expression. **d** Hierarchical clustering heatmap (left) and results of KEGG pathway enrichment analysis (right) of the differentially expressed genes from the PBMCs of the DIC and WT pigs after administration of Dox for 0 and 14 days. **e** Heatmap showing the relative expression of genes related to genomic damages, apoptosis, cell senescence and the P53 signaling pathway. The Z score was calculated to indicate relative expression between samples. **f** The bar plot shows the number of indels and SNVs detected in the PBMCs of DIC and WT pigs after administration of Dox for 14 days, with background mutations excluded. Data are presented as mean ± SEMs. **g** The Circos plot shows the whole genome distributions of the detected mutations in the PBMCs of DIC and WT pigs

Next, to investigate the effects of Cas9 expression in vivo, we examined transcriptome homeostatic changes and genome mutation enrichments in the PBMCs of DIC pigs. Transcriptome sequencing was performed on PBMCs collected from four DIC pigs and three age-matched WT pigs on Day 0 and Day 14 of Dox administration (referred to as DIC-D0, DIC-D14, WT-D0, and WT-D14, respectively) (Fig. 2a). To explore the global transcriptional changes after administration with Dox for 14 days, we grouped DIC (DIC-D0 and DIC-D14) and WT (WT-D0 and WT-D14) samples in a principal component analysis (PCA) space. PCA confirmed that obvious transcriptional changes were found in principal component 1, which accounted for 52% of the sample variations, whereas no visible transcriptional changes occurred in the space of principal component 2, which accounted for 10% of the sample variations (Fig. 2b). These results indicated that the expression profiles of DIC-D0 were similar to those of WT-D0 and WT-D14, whereas the expression profiles of DIC-D14 obviously changed. Additionally, hierarchical clustering analysis demonstrated that the gene expression patterns of DIC-D0 were similar to those of WT-D0 and WT-D14, while DIC-D14 samples were distinct from the other groups (Supplementary Fig. 6). Differential expression analysis comparing DIC-D14 with DIC-D0 revealed that Cas9 expression upregulated 2,003 genes and downregulated 1,730 genes after adjusting for the influence of Dox (WT-D14 compared to WT-D0 was used as the control) (Fig. 2c). These findings indicated that Cas9 expression induced changes in the global transcriptional profiles of PBMCs in vivo. Furthermore, KEGG pathway enrichment analysis demonstrated that the 3,733 differentially expressed genes (DEGs) were primarily enriched in DDR-related pathways, including cellular senescence, ubiquitin-mediated proteolysis, the P53 signaling pathway, apoptosis, proteasome and mitophagy, as well as immune-related pathways, such as Th17 cell differentiation, haematopoietic cell lineage, the B-cell receptor signaling pathway, the T-cell receptor signaling pathway, and Th1 and Th2 cell differentiation (Fig. 2d). Detailed expression analysis of apoptosis-multiple species, apoptosis, cell senescence, and P53 signaling pathway genes in the DIC-D14 groups compared to the DIC-D0, WT-D0 and WT-D14 groups. The results showed that the DIC-D14 groups exhibited specific activation of apoptosis-multiple species, cell senescence, and P53 signaling pathway genes, while apoptosis genes were specifically depressed (Fig. 2e). Furthermore, we observed activation of the *MRE11*, *ATR*, *ATM*, and *RAD1* genes in the DIC-D14 groups, providing further evidence that Cas9 induced genomic damages in DIC pigs.

To investigate whether genome mutations were enriched in PBMCs, we performed WGS of PBMCs from two DIC pigs on days 0 and 14 of Dox administration, with two WT pigs serving as controls (Fig. 2a). Somatic mutations in PBMCs were detected using the mutation caller Strelka2.^38^ Overall, the number of genomic mutations in the PBMCs of the DIC pigs was slightly higher than that in the PBMCs of the WT pigs, but the difference was not statistically significant. Specifically, we identified a total of 1,719 indels and 6,267 single-nucleotide variants (SNVs) in the PBMCs of the DIC pigs, while in comparison, the PBMCs of the WT pigs also displayed comparable detectable mutations, with 1,451 indels and 5,737 SNVs (Fig. 2f). Additionally, a Circos plot was generated to visualize the distribution of mutations across the entire genome. The results showed that these indels and SNVs were randomly distributed among the chromosomes, with no discernible differences between DIC and WT pigs (Fig. 2g). These findings suggest that Cas9 expression for two weeks in the PBMCs of DIC pigs did not increase the number of genomic mutations.

### Changes in global gene expression patterns in the solid organs of DIC pigs with Dox administration

**Fig. 3:**
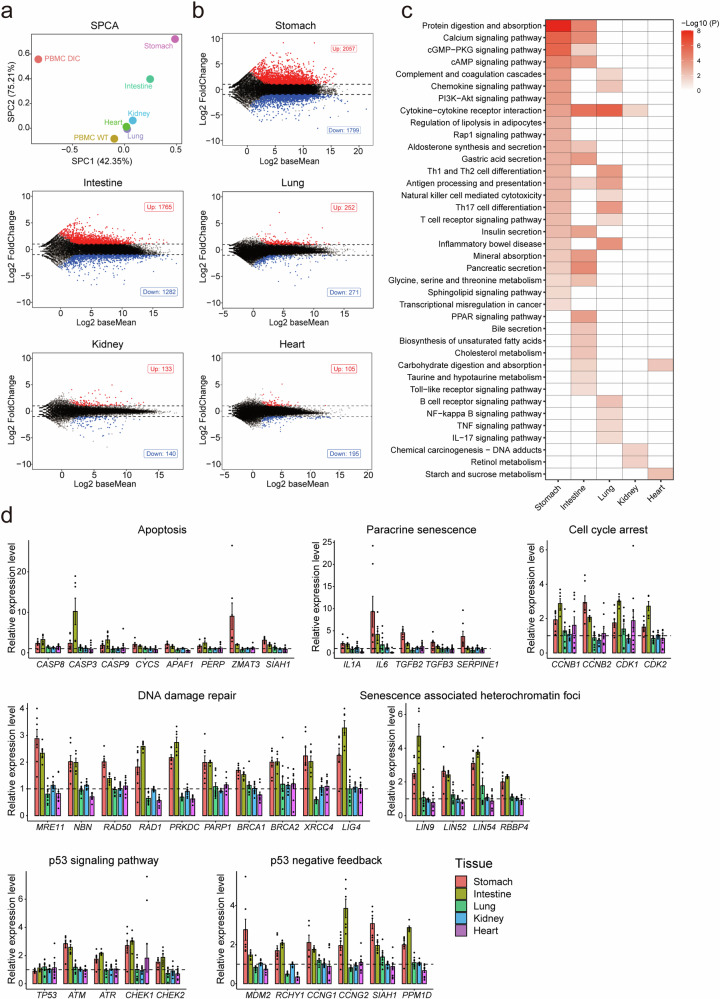
Transcriptome sequencing analysis of the solid organs of DIC pigs after two weeks of Dox administration. **a** Sparse principal component analysis (SPCA) of the solid organ samples by the log2FoldChange values of the whole transcriptome. **b** MA plot showing the up- and downregulated differentially expressed genes in the stomach, intestine, lungs, kidneys, and heart of the DIC pigs after the administration of Dox for 14 days. **c** KEGG pathway enrichment analysis of the differentially expressed genes in the solid organs of the DIC pigs after administration of Dox for 14 days. **d** Bar plot of DDR-related pathway genes in the different solid organs. Data represent the means, and error bars correspond to SEMs

To further explore the changes in biological pathways in the solid organs caused by Cas9 expression, we performed transcriptome sequencing of the stomach, intestine, lungs, kidneys, and heart tissues of three DIC pigs and three age-matched WT pigs with Dox administration. PCA demonstrated that the transcriptional profiles of the tissues of the DIC pigs were distinct from those of the tissues of the WT pigs (Supplementary Fig. 7). Additionally, we compared the transcriptional profiles of DIC pigs with those of WT pigs for each tissue. The global transcriptional profiles of the lungs, kidneys, and heart had fewer fluctuations and were closer to the global transcriptional profiles of PBMCs in WT-D14 compared to WT-D0 (PBMC-WT). On the other hand, the transcriptional profiles of the stomach and intestine were more similar to those of the PBMCs in DIC-D14 compared to DIC-D0 (PBMC-DIC), with greater fluctuations in the SPCA space (Fig. 3a). Further analysis of the transcriptional profiles of the tissues of the DIC pigs compared to those of the tissues of the WT pigs showed a positive correlation between the number of DEGs and the protein expression levels of Cas9 in the solid organs (Fig. 3b, Supplementary Fig. 2b). Specifically, the stomach and intestine displayed 4,306 DEGs (2,057 upregulated and 1,799 downregulated) and 3,047 DEGs (1,765 upregulated and 1,282 downregulated), respectively (Fig. 3b). However, only 523 DEGs (252 upregulated and 271 downregulated), 273 DEGs (133 upregulated and 140 downregulated), and 300 DEGs (105 upregulated and 195 downregulated) were detected in the lungs, kidneys, and heart, respectively (Fig. 3b). These findings further support the notion that Cas9 expression affects the global transcriptional profiles of solid organs and that the extent of this impact is positively correlated with the Cas9 expression level.

We then performed KEGG pathway enrichment analysis of the DEGs to explore changes in biological pathways caused by Cas9 expression in these organs (Fig. 3c). The results revealed the enrichment of signaling pathways related to stomach and intestine function, such as protein digestion and absorption, the regulation of lipolysis in adipocytes, aldosterone synthesis and secretion, gastric acid secretion, mineral absorption, and glycine, serine, and threonine metabolism. These findings suggest that the function of the stomach and intestine may have been altered. Interestingly, we did not observe enrichment of the senescence, apoptosis and P53 signaling pathways in these DEGs of the stomach and intestine in DIC pigs with Dox administration for two weeks. This may be attributed to the short duration of Cas9 expression, which did not induce the enrichment of these pathway genes.

To elucidate whether Cas9 had an impact on these signaling pathways in tissues, we evaluated the activation of key genes involved in apoptosis, cellular senescence, the cell cycle, DNA damage repair, and the P53 signaling pathway in different organs (Fig. 3d). Our results revealed a significant increase in the activation of genes associated with apoptosis (*CASP8*, *CASP3*, *CASP9*, *CYCS*, *APAF1*, *PERP*, *ZMAT3*, and *SIAH1*), paracrine senescence (*IL1A*, *IL6*, *TGFB2*, *TGFB3*, and *SERPINE1*), cell cycle arrest (*CCNB1*, *CCNB2*, *CDK1*, and *CDK2*), DNA damage repair (*MRE11*, *NBN*, *RAD50*, *RAD1*, *PRKDC*, *PARP1*, *BRCA1*, *BRCA2*, *XRCC4*, and *LIG4*), and senescence-associated heterochromatin foci (*LIN9*, *LIN52*, *LIN54*, and *RBBP4*) in the stomach and intestine of DIC pigs compared with WT pigs. Although *P53* was not highly expressed in the stomach or intestine, the activation of downstream genes (*ATM*, *ATR*, *CHEK1*, and *CHEK2*) and P53 negative feedback genes (*MDM2*, *RCHY1*, *CCNG1*, *CCNG2*, *SIAH1*, and *PPM1D*) was significantly increased in these organs.

Our findings indicate that organs with high expression of the Cas9 protein, such as the stomach and intestine, exhibited more severe genomic damages and were more likely to demonstrate changes in global gene expression patterns, leading to increased apoptosis and/or senescence compared to that in other organs. In contrast, organs with low expression of the Cas9 protein, such as the lungs and kidneys, as well as those that rarely expressed the Cas9 protein, such as the heart, did not show obvious genomic damages and did not exhibit changes in gene expression patterns.

### The prolonged expression of Cas9 resulted in abnormal phenotypes in DIC pigs

**Fig. 4:**
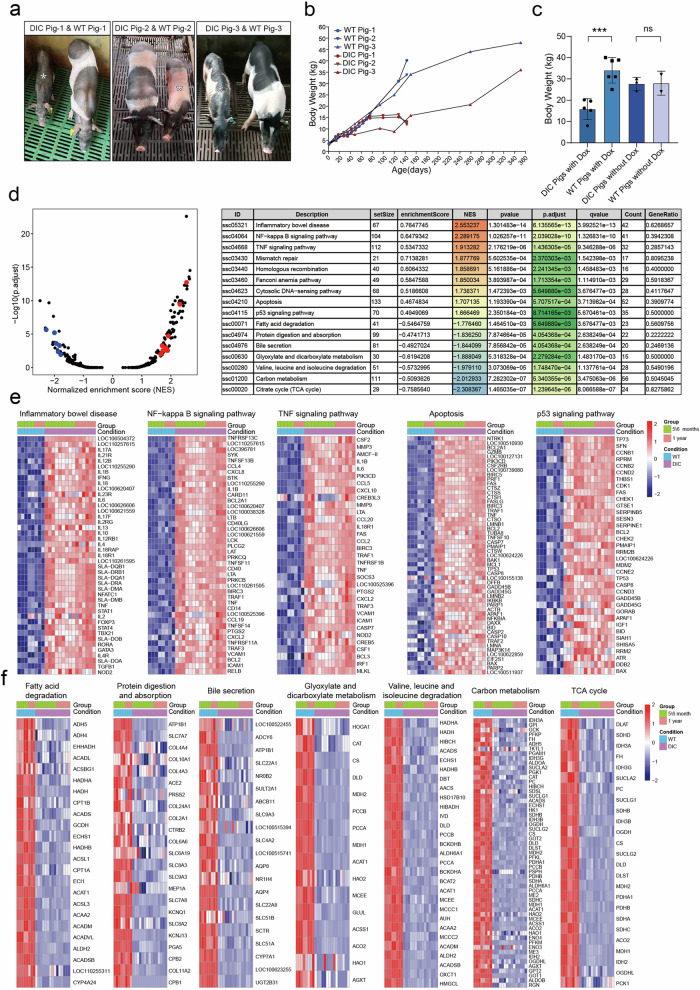
The phenotypes of DIC pigs with continuous expression of Cas9 were analyzed. **a** Images of DIC pigs and age-matched WT pigs after long-term Cas9 expression. **b** The growth curves of DIC pigs (DIC Pig-1, DIC Pig-2 and DIC Pig-3) and age-matched WT pigs (WT Pig-1, WT Pig-2 and WT Pig-3) fed Dox. **c** The bar chart shows the results of statistical analysis of the body weights of DIC pigs treated with Dox, WT pigs treated with Dox, DIC pigs without Dox treatment and WT pigs without Dox treatment for 120~135 days. ***p = 0.0004, unpaired *t* test. Data are presented as mean ± SEMs. **d** The dot plot (left) and detailed table (right) shows the results of GSEA of stomach samples from the DIC pigs after Dox feeding. **e**, **f** Heatmap showing the relative expression of genes in activated and depressed signal pathways in stomach samples. The Z score was calculated to indicate relative expression between samples

Since Cas9 expression for two weeks affected the signaling pathways related to stomach and intestine function, we next explored whether prolonged Cas9 expression would cause abnormal phenotypes in the DIC pigs. We administered Dox to five DIC pigs and six age-matched WT pigs (pigs with knock-in at only one locus or without knock-in at any loci). Four DIC pigs (DIC pig-1, DIC pig-2, DIC pig-7, and DIC pig-8) and five WT pigs (WT pig-1, WT pig-2, WT pig-6, WT pig-7, and WT pig-8) received daily oral administration of Dox, while DIC pig-3 and WT pig-3 received Dox both orally and by intraperitoneal injection for the first month, followed by daily oral administration. Flow cytometry analysis confirmed that the Cas9 expression was induced in DIC pigs by Dox (Supplementary Fig. 8a). As expected, with continuous Dox administration, the DIC pigs exhibited significantly lower body weight compared to that of the WT pigs (Fig. 4a–c, Supplementary Fig. 8d). The monitoring of body weight of the DIC and WT pigs revealed that DIC pig-1 and DIC pig-2 started retarded growth at 70 days post oral Dox administration, DIC pig-7 and DIC pig-8 initiated weight reduction at 33 days post oral Dox administration, while DIC pig-3 began growth retardation at 28 days post Dox administration (Fig. 4b, Supplementary Fig. 8d). The body weight of DIC pig-3 was only one-third that of WT pig-3 after 120 days of Cas9 expression. The severe weight loss correlates with the higher Cas9 induction efficiency observed in DIC pig-3 during the first month (Supplementary Fig. 8a). To ensure animal welfare, Dox administration in the two pigs was stopped for one month. During this period, the body weight of DIC pig-3 increased again. To further confirm that these abnormal phenotypes were caused by the expression of Cas9 protein, we also monitored the body weight of three DIC pigs (DIC Pig-4, DIC Pig-5, and DIC Pig-6) and two age-matched WT pigs (WT Pig-4 and WT Pig-5) without Dox administration. The expression of Cas9 protein was not detected in DIC pigs without Dox treatment, and there was no significant difference in body weight between these DIC pigs and WT pigs (Fig. 4c, Supplementary Fig. 8b, c). We further analyzed the body weight of these five DIC pigs and six WT pigs administrated with Dox, and three DIC pigs and two WT pigs administrated without Dox on 120-135 days (120 days: WT Pig-4, WT Pig-5, DIC Pig-4, DIC Pig-5, DIC Pig-6; 125 days: WT Pig-3, DIC Pig-3; 128 days: WT Pig-1, WT Pig-2, DIC Pig-1, DIC Pig-2; 135 days: WT Pig-6, WT Pig-7, WT Pig-8; DIC Pig-7, DIC Pig-8) (Fig. 4b, Supplementary Fig. 8c, d). The results showed that the body weight of the DIC pigs treated with Dox was significantly lower than that of the WT pigs treated with Dox, while the body weight of the DIC pigs without Dox treatment was similar to that of the WT pigs with/without Dox treatment (Fig. 4c). In the first month of Dox-induced Cas9 expression, we observed that all DIC pigs treated with Dox exhibited diarrheal symptoms, while no diarrhea was found in DIC pigs without Dox treatment and the WT pigs with/without Dox treatment.

To evaluate whether prolonged expression of Cas9 protein could result in transcriptome changes and enrichment of DNA mutations, five DIC pigs and three age-matched WT pigs were euthanized after 5/6 months or 1 year of Dox administration (Group 1: DIC pig-1, DIC pig-2, WT pig-1, and WT pig-2: 5 months Dox administration; DIC-7: 6 months Dox administration; Group 2: DIC pig-3, DIC pig-8, and WT pig-3: 1 year Dox administration). Consistent with previous experiments, Cas9 protein was highly expressed in the stomach and intestine, moderately expressed in the lungs, and expressed at low levels in the heart (Supplementary Fig. 8e). Genomic damages were evident in the stomach, intestine, and lungs, while no change in the heart, as demonstrated by IF labeling of pH2AX (Supplementary Fig. 9a) and TUNEL staining (Supplementary Fig. 9b). The collected tissues of stomach, intestine, lungs, and heart from DIC pigs and WT pigs with long-term Dox administration were performed transcriptome sequencing. Transcriptome sequencing analysis revealed that a significant increase in the activation of DDR-related signaling pathways, including inflammatory bowel disease, the NF-kappa B signaling pathway, the TNF signaling pathway, apoptosis, and the P53 signaling pathway, in the stomach and intestine of DIC pigs with long-term expression of Cas9 protein (Fig. 4d, Supplementary Fig. 10a). In addition, mismatch repair, homologous recombination, the Fanconi anemia pathway, and the cytosolic DNA-sensing pathway were activated in the stomach, and cellular senescence was activated in the intestine (Fig. 4d, Supplementary Fig. 10a). Conversely, signaling pathways involved in fatty acid degradation, protein digestion and absorption, bile secretion, glyoxylate and dicarboxylate metabolism, valine, leucine, and isoleucine degradation, carbon metabolism, and the TCA cycle were significantly suppressed in the stomach (Fig. 4d). Furthermore, signaling pathways related to glutathione metabolism, porphyrin metabolism, steroid hormone biosynthesis, tyrosine metabolism, glycine, serine, and threonine metabolism, and retinol metabolism were significantly suppressed in the intestine (Supplementary Fig. 10a). The major genes involved in inflammatory bowel disease, the NF-kappa B signaling pathway, the TNF signaling pathway, apoptosis, and the P53 signaling pathway were consistently activated in the stomach, intestine, and lungs (Fig. 4e, Supplementary Fig. 10b, c). However, no signaling pathway showing obvious activation was observed in the heart (Supplementary Fig. 10d). In contrast to the results observed after two weeks of Cas9 expression, *P53* gene activation was detected in all tested tissues, suggesting that Cas9 activates the P53 signaling pathway, with the degree of activation depending on the time and level of Cas9 expression. The cytosolic DNA-sensing pathway was also enriched and activated in the stomach of DIC pigs, likely due to Cas9-induced genomic damages, which resulted in fragmented DNA in the cytoplasm. Fatty acid degradation, protein digestion and absorption, bile secretion, glyoxylate and dicarboxylate metabolism, valine, leucine, and isoleucine degradation, carbon metabolism, and TCA cycle signaling pathway genes were suppressed in the stomach, suggesting digestive dysfunction in the DIC pigs (Fig. 4f). IHC staining showed that CD68-positive inflammatory cells were enriched in the stomach, intestine, and lung tissues of DIC pigs, but rarely observed in the heart of DIC pigs or in WT pigs, proving that DIC pigs indeed experienced inflammatory impairment (Supplementary Fig. 10e). Western blotting exhibited the elevated expression of ATM (the DNA damage response messenger protein) and CASP3 (the cell apoptosis effector protein) in the stomach and intestine tissues of DIC pigs, suggesting that severe genomic damages and cellular apoptosis occurred in the stomach and intestine tissues of DIC pigs (Supplementary Fig. 10f). We could not find the increased expression of ATM and CASP3 in the lungs of DIC pigs, which could be due to the relative lower expression of Cas9 in the lung tissues. These results were consistent with the results of transcriptome sequencing in Fig. 3d. We proposed a hypothesis underlying the weight loss observed in the DIC pigs: long-term overexpression of Cas9 directly caused genomic damages in the stomach and intestine, activating the P53 signaling pathway and leading to senescence, apoptosis, and ultimately, inflammatory bowel disease. These processes affected the absorption and metabolic functions of the stomach and intestine, leading to digestive dysfunction and slow weight gain in DIC pigs.

### Genome-wide mutations increased with long-term Cas9 expression in DIC pigs

**Fig. 5:**
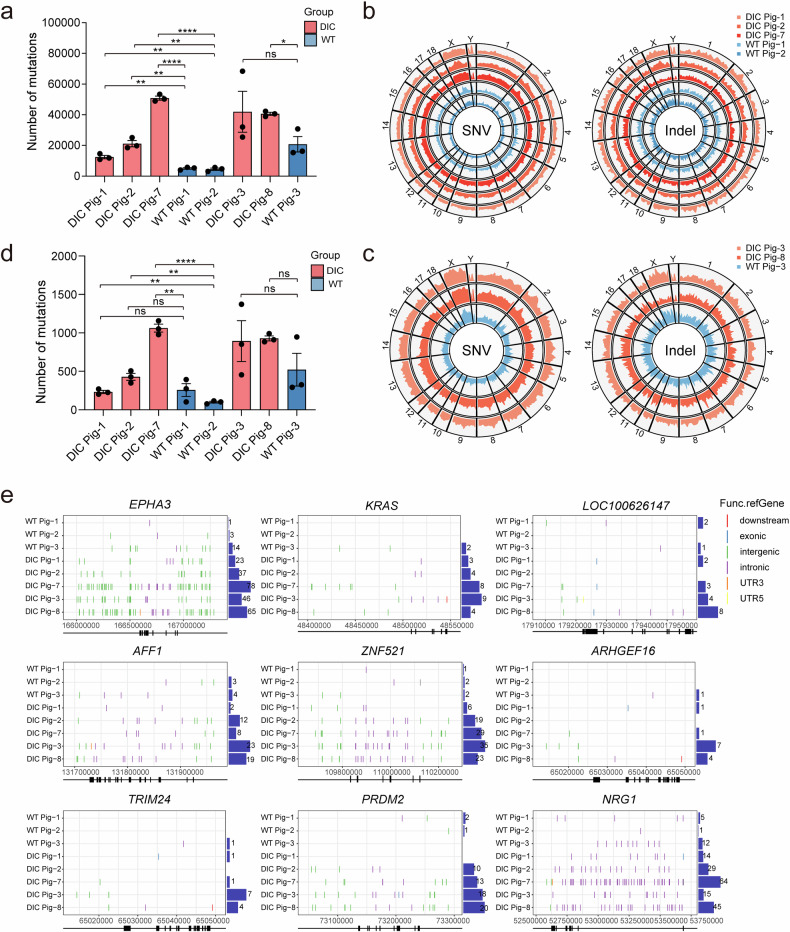
Whole-genome sequencing analysis of DIC pigs with continuous expression of Cas9. **a** The bar plot shows the number of genome mutations detected in stomach samples from DIC pigs and WT pigs after prolonged dosing of Dox. Data represent the means, and error bars correspond to SEMs. **b**, **c** The Circos plot shows the whole-genome distributions of the detected mutations in stomach samples from the DIC and WT pigs with long-term Cas9 expression. The height of each track represents the mutation density at a window size of 10 million bases. **d** The bar plot shows the number of mutations on tumor driver genes in stomach samples from the DIC and WT pigs with long-term Cas9 expression. Data represent the means, and error bars correspond to SEMs. **e** The type and location of mutations in some tumor driver genes in the stomach of DIC and WT pigs with long-term Cas9 expression are shown

To evaluate whether genomic mutations were enriched in DIC pigs with long-term Cas9 expression, we performed comparative WGS of gDNA extracted from the tissues with the highest (stomach) and lowest (heart) Cas9 expression in DIC pigs. For the group of 5/6 months Dox administration, we detected a total of 12,482 (1,262 indels and 11,220 SNVs), 21,153 (1,808 indels and 19,345 SNVs), and 50,878 (1,641 indels and 49,237 SNVs) mutations in DIC Pig-1, DIC Pig-2, and DIC Pig-7, respectively (Fig. 5a, Supplementary Fig. 11a). In comparison, 5,044 (689 indels and 4,355 SNVs) and 4,561 (596 indels and 3,965 SNVs) mutations were observed in the genomes of WT pig-1 and WT pig-2, respectively. The number of genome mutations in the DIC pigs were significantly higher than those in the WT pigs (Fig. 5a). For the group of 1 year Dox administration, 41,977 (2,274 indels and 39,703 SNVs) and 40,599 (1,200 indels and 39,399 SNVs) mutations were observed in the genomes of the DIC pig-3 and DIC pig-8, respectively, which were higher than WT pig 3 (20,822 mutations: 1,930 indels and 18,892 SNVs) (Fig. 5a, Supplementary Fig. 11a). Chromosome distribution analysis revealed no specific enrichment of mutated genes in any chromosome in the tissues with long-term Cas9 expression (Fig. 5b, c), consistent with the distribution of genome mutations observed in PBMCs of DIC pigs after two weeks of Cas9 expression (Fig. 2G). These results suggest that Cas9 increases mutations and genomic damages across all chromosomes. Genome mutations typically induce cell abnormalities and cell death and can even trigger cancer development in vivo. To determine whether long-term Cas9 expression increased the occurrence of tumors in pig models, we compared and analyzed mutations in 568 tumor driver genes in the genomes of DIC pigs and WT pigs.^39^ The number of tumor driver gene mutations in the DIC pig genome was higher than that in the WT pig genome. Among these DIC pigs, 233 (24 indels and 209 SNVs), 427 (37 indels and 390 SNVs), 1,063 (33 indels and 1,030 SNVs), 893 (46 indels and 847 SNVs), and 929 (29 indels and 900 SNVs) mutations were observed in DIC Pig-1, DIC Pig-2, DIC Pig-7, DIC Pig-3 and DIC Pig-8, respectively, while 257 (18 indels and 239 SNVs), 99 (13 indels and 86 SNVs), and 522 (44 indels and 478 SNVs) mutations were observed in WT Pig-1, WT Pig-2, and WT Pig-3, respectively (Fig. 5d, Supplementary Fig. 11b). The mutations were mainly observed in intergenic and intronic regions, with only a few occurring in downstream, exonic, UTR3, or UTR5 regions of the genes. Examples included the mutations of the region downstream of *KRAS*, *ARHGEF16*, and *TRIM24*, the mutations of *EPHA3*, *AFF1*, and *NRG1* in the UTR3, and the mutations of *LOC100626147* in the UTR5. The mutations of *LOC100626147*, *ARHGEF16*, *TRIM24*, *PRDM2*, and *NRG1* genes were observed in the exons (Fig. 5e). The mutations in *LOC100626147* locus caused an 8-bp deletion (g.17926827_17926834 delCACGTCGA) in DIC Pig-1, synonymous variant (g.17926874G>A) in DIC Pig-7, and nonsynonymous variant (g.17925880T>C and g. 17925904C>T, resulting in p.Val999Ala and p.Ala1007Val) in DIC Pig-8. The mutations in *ARHGEF16* gene, a 1-bp deletion (g.65035338 delG) in DIC Pig-1, resulted in the presence of premature termination codons. The mutations in *NRG1* gene caused a 3-bp deletion (g.53687437_53687439 delCCG) in DIC Pig-1, which was a non-frameshift mutation. The mutations in *TRIM24* gene was a synonymous variant (g.52667A>G), and in the *PRDM2* gene, it was a nonsynonymous variant (g.73205051T>C), resulting in p.Leu1221Pro in DIC Pig-3. Despite the absence of tumor formation in any of the five DIC pigs, the expression of Cas9 protein led to an increase in genome-wide mutation rates. This surge in mutations unfortunately included alterations in several key tumor-driving genes, which, in turn, heightened the susceptibility of cancer development.

## Discussion

In this study, we investigated the safety of guide-free Cas9 for gene therapy using our unique Dox-inducible Cas9-expressing pig model. Previous studies in which only immortalized cell lines and stem cells were tested have shown that the expression of Cas9 alone can lead to genomic toxicity in vitro.^29,30^ Our study, in which somatic cells, the major targeted cells for gene therapy, were tested, is the first to demonstrate this effect in vivo. We observed an increase in pH2AX expression, as well as the presence of nuclear comet tails, both of which are indicators of genomic damages.

For in vivo gene therapy, one common approach is to deliver Cas9 expression vectors through vein injection.^9,34,40^ Therefore, we first evaluated whether guide-free Cas9 could induce genomic damages in the PBMCs of our DIC pigs after Dox induction. We observed an elevation in the pH2AX-positive cell number and the formation of nuclear comet tails, indicating genomic damages in PBMCs. We also investigated whether Cas9 could cause genomic damages in solid organs, as direct injection into organs is another approach for in vivo gene therapy.^35,41^ We observed that the expression levels of Cas9 varied among different organs following Dox treatment, with the highest expression levels observed in the stomach and intestine. This was followed by the lungs and kidneys, while the heart exhibited the lowest expression levels. In alignment with the expression levels, we noted that the severity of genomic damages was greatest in the stomach and intestine, moderate in lungs and kidneys, and mildest in the heart (comparable to that of WT pigs). Furthermore, the detrimental effects of Cas9 were positively correlated with its expression levels, with organs exhibiting high levels of Cas9 expression being more susceptible to increased apoptosis and/or senescence. Additionally, we investigated the relationship between genomic damages and the duration of Cas9 expression in PBMCs. Our findings revealed that as the duration of Cas9 expression increased, the severity of genomic damages also increased.

Transcriptome changes were also observed in the PBMCs and solid organs (stomach and intestine) of DIC pigs expressing Cas9, indicating a clear change in gene expression patterns. These changes were particularly enriched in pathways related to the DDR, consistent with previous studies suggesting that genomic damages trigger the activation of DDR to repair DNA damages.^42^ In line with these findings, the expression of genes involved in DDR was specifically increased in both the PBMCs and organs with high Cas9 expression (stomach and intestine).

Severe DNA damage can lead to cell apoptosis or senescence,^43^ and therefore, it is not surprising that the activation of major genes related to apoptosis and cellular senescence was also observed in PBMCs, the stomach, and the intestine. Consistent with previous reports,^29,30^ the P53 signaling pathway was also activated upon the induction of Cas9 expression in vivo, as evidenced by the upregulation of genes downstream of *P53* in PBMCs and solid organs. Interestingly, KEGG pathway enrichment analysis revealed that the P53 signaling pathway was enriched specifically in the stomach of DIC pigs with long-term Cas9 expression, but not in the stomach of DIC pigs with two weeks of Cas9 expression. This finding suggests that the activation level of the P53 signaling pathway in vivo may be influenced by the duration of Cas9 expression.

Based on these results, it can be inferred that limiting the expression level of Cas9 within a specific time frame after its gene-correcting function has been fulfilled could help to mitigate its detrimental effects. This can be achieved by inhibition of CRISPR/Cas9 with bacteriophage proteins^44^ or small molecules,^45^ application of modified versions of Cas9 with fast turnover^46^ or chemically controlled split-Cas9^47^ during gene editing practice. Additionally, administration of drugs targeting DNA repair pathways when delivering CRISPR/Cas9 in vivo should be an alternative way to reduce its adverse effects.^48^

Furthermore, we also observed an adverse phenotype in DIC pigs with long-term Cas9 expression, indicating a detrimental effect of Cas9 that warrants serious consideration. Unlike transgenic mice expressing cytosine base editors,^49^ our DIC pigs exhibited increased weight loss compared to that of the control group. This difference could be attributed to variations in the manner of Cas9 expression and the abundance of Cas9 expressed in different organ tissues. Through transcriptome sequencing analysis, we observed the upregulation of inflammatory genes in the stomach and intestine of DIC pigs. In contrast, the expression of genes related to lipid, protein and carbon metabolism was downregulated. Additionally, the activation of DDR-related signaling pathways, as well as the NF-kappa B and TNF proinflammatory signaling pathways, was observed. Based on these findings, we speculate that the weight loss seen in DIC pigs is the result of persistent Cas9-induced genomic damages in the stomach and intestine, leading to the activation of the NF-kappa B and TNF proinflammatory signaling pathways. This process ultimately causes inflammation and dysfunctions in nutrient absorption.

These in vivo results from pigs corroborate in vitro observations in porcine embryos,^31^ where the expression of the Cas9 protein has been shown to induce genomic damages and transcriptome changes within the blastocyst. These transcriptome changes include the depression of embryo development signaling pathways and activation of apoptosis and necroptosis signaling pathways, leading to impaired preimplantation embryonic development. The evaluation of detrimental effects caused by Cas9 protein not only for preimplantation embryos in vitro but also for somatic cells in vivo provided the safety concerns for both CRISPR/Cas9-mediated germline and somatic gene therapy for human genetic disorders.

In conclusion, this study is the first time to comprehensively evaluate the detrimental effects of Cas9 protein in large animals in vivo. Our investigations revealed that the expression of Cas9 protein in pigs significantly increased genomic damages, induced transcriptome changes, and enriched genomic mutations in the DIC pigs. Notably, prolonged expression of Cas9 protein resulted in growth retardation and alterations of key tumor-driving genes. Although our study did not observe direct tumor development, it’s crucial to highlight the increased tumor risk potentially associated with extended Cas9 protein expression. This highlights another important risk factor that needs to be considered in clinical trials for gene therapy.

## Materials and methods

### Ethics statements

All the animal experiments in this study were approved by the Institutional Animal Care and Use Committees (IACUC) at Guangzhou Institute of Biomedicine and Health, Chinese Academy of Sciences (certificate number, N2021041 and N2021110).

### Animals

DIC pigs used in this study were previously established by our group, which integrated the rtTA expression cassette and TRE3G-Cas9-T2A-tdTomato cassette into the porcine endogenous *Rosa26* and *Hipp11* loci.^37^ The pigs used in this study were housed under standard conditions in the joint large animal facility of Guangzhou Institute of Biomedicine and Health, Chinese Academy of Sciences and Wuyi University.

In this study, we started to feed Dox to DIC and WT piglets with 2~5 weeks old. According to the standard dosage of Dox (50 mg/kg/day), the pigs were administered daily oral and/or intraperitoneal injection every two days. Four DIC pigs and three WT pigs of the same age were selected with Dox administration for two weeks. In addition, five DIC pigs and six age-matched WT pigs were fed with Dox for 5/6 months or 1 year. Three DIC pigs and two WT pigs without Dox administration were used as negative control. The information of these pigs was shown in Supplementary Table 1. Primers used for genotyping of DIC pigs were listed in Supplementary Table 2.

### Collection and isolation of PBMCs

Blood was collected from the anterior vena cava of pre-anesthetized pigs and stored in vacuum anticoagulant tubes. The collected pig blood was centrifuged at 500 × *g* for 5 min, the upper serum was aspirated, and 10 times the volume of red blood cell lysis buffer (Beyotime, C3702) was added and incubated at room temperature for 10 min to lyse the red blood cells. The obtained PBMCs were then subjected to further experiments.

### Tissue sample collection and preservation

The DIC pigs and WT pigs were euthanized by exsanguination after anesthesia. Each type of pig tissue (heart, lungs, kidneys, stomach, and intestine) was collected and then stored in a liquid nitrogen tank or 4% paraformaldehyde for further use.

### Flow cytometry analysis

PBMCs after red blood cell lysis were prepared into single-cell suspensions and directly subjected to flow cytometry to detect tdTomato expression. For the detection of Cas9, the first antibody used in this study was anti-CRISPR‒Cas9 (Huabio, ET1703-85), and the second antibody was anti-rabbit IgG(H + L) (CST, 4412S). The stained samples were kept on ice and analyzed by flow cytometry as quickly as possible. For each sample, 10,000–20,000 live cells were recorded.

### IF staining and fluorescence analysis

After red blood cell lysis, PBMCs were seeded in poly-L-lysine-coated cover glasses, fixed with 4% paraformaldehyde for 15 min and permeabilized with 9% Triton-X for 1 h. IF staining was performed using primary antibodies to detect pH2AX (CST, 9718). The nuclei were stained with DAPI (Sigma‒Aldrich, F6057) for 10 min. Confocal image acquisition was performed using a Zeiss LSM 710 laser-scanning microscope. Each porcine tissue sample stored in a liquid nitrogen tank was frozen-sectioned, and subsequent IF staining was performed according to the operation described above to detect pH2AX and Casp3 (CST, 9661). We used a TUNEL apoptosis detection kit (Vazyme, A111) to analyze the apoptosis in each porcine tissue.

### Comet assay

Blood was collected from the anterior vena cava of pre-anesthetized pigs, washed three times with ice-cold PBS, resuspended at a density of 10^6^ cells/mL mixed with low-melting agarose at a ratio of 1:3, and then spread onto glass slides. The prepared slides were protected from light and incubated at 4 °C for 15 min. The slides were soaked in frozen lysis solution for 60 min, and then washed three times with frozen PBS. Under dark conditions, the slides were electrophoresed in precooled TBE buffer. Subsequently, the slides were stained with Vista Green DNA dye (Solarbio, G8140) and then observed under an inverted fluorescence microscope. The results were analyzed by the Comet Assay Software Project.

### RNA extraction and quantitative PCR

Total RNA was extracted from each sample by using an RNeasy Mini Kit (QIAGEN, 74104). First-strand cDNA was synthesized using a StarScript II cDNA Kit (Genstar, A224-10) in accordance with the manufacturer’s instructions. Following the manufacturer’s instructions, fluorescence quantitative PCR was performed with AceQ® Universal SYBR® qPCR Master Mix (Vazyme, Q511-02). Primers used in this study were listed in Supplementary Table 2.

### Western blotting

Tissues were collected from the organs of DIC pigs and WT pigs. Approximately 20–50 mg of tissues were grinded to extract total protein by using RIPA (Thermo, 89901) and protease inhibitor (Thermo, 87785). BCA Protein quantification kit (Genstar, E16201) was used to assay the concentration. The total protein was used to perform SDS-PAGE and transferred to PVDF membrane. The membrane was incubated with a 5% BSA blocking buffer for 2 h and incubated with ATM (Proteintech, 67586), CASP3 (Proteintech, 66470) and GAPDH (Beyotime, AF2823) antibody, separately. After washing three times with TBST buffer, the PVDF membrane was incubated with HRP-Goat anti-Rabbit IgG or HRP-Goat anti-Mouse IgG at room temperature for 2 h, and then washed with TBST three times. Finally, the targeted protein was detected by using ECL hypersensitive luminescence kit (Vazyme, E411) and MiniChemi^TM^ 830.

### IHC staining

Fresh tissue samples from dissected pigs were collected and soaked in 4% paraformaldehyde at room temperature for 24–48 h, then embedded in paraffin, and finally sectioned. Dried slices were dehydrated with xylene and different concentrations of ethanol. Antigen recovery was conducted in citrate buffer at 100 °C for 10 min and cooled to room temperature. Next, 3% hydrogen peroxide was applied to the section to block peroxidase. After washing with PBS, slices were blocked with the goat serum for 1 h. Then, they were incubated with Cas9 or CD68 antibody overnight at 4 °C in a wet box and then incubated with HRP-conjugated Rabbit anti-goat IgG antibody on the second day. Finally, the slices were treated with DAB and sealed with neutral gum for observation.

### Transcriptome sequencing

The freshly collected PBMCs and tissues were stored in RNAlater stabilization solution (Thermo Fisher, AM7020) at −80 °C before total RNA was extracted using TRIzol (Thermo Fisher). A library was constructed with a VAHTS mRNA-seq V3 Library Prep Kit for Illumina (Vazyme) in accordance with standard instructions. The libraries qualified by Agilent 2100 were sent to Annoroad Gene Technology Corporation (Beijing) for next-generation sequencing. Different samples were distinguished by the addition of a unique index into adapter sequences upon library preparation. The library was sent to GENEWIZ and HAPLOX for sequencing on a NovaSeq instrument.

### Transcriptome sequencing data analysis

Raw reads were processed by fastp (version 0.20.1)^50^ to filter out low quality reads and remove sequencing adapters. Then, gene expression levels were quantified by salmon (version 1.4.0)^51^ using pig transcripts defined by NCBI (GCF_000003025.6). The R package DESeq2 (version 1.38.1)^52^ was used to perform differential expression analysis. For PBMCs, we set day 0 as the control group and day 14 as the experimental group. To diminish the noise introduced by Dox, we excluded the DEGs in DIC pigs on day14 which also represented in WT pigs on day14. For stomach, intestine, lungs, kidneys, and heart, we collected corresponding tissue samples from WT individuals born in the same litter as controls, and these WT individuals were also treated with Dox. In each group, DEGs were defined as genes for which |log2FoldChange| > 1 and *p*-adjusted-value < 0.05. Sparse principal component analysis (SPCA) was performed with Log2FoldChange values using the SPC function from the R package PMA (version 1.2.1).^53^ Gene enrichment analysis was performed by the R package clusterProfiler (version 4.6.2).^54^ Scatterplots and bar plots were constructed using the R package ggplot2 (version 3.4.0) and custom scripts in R. A heatmap was constructed using the R package pheatmap (version 1.0.12).

### WGS data analysis

Raw reads were processed by fastp (version 0.20.1)^50^ to filter out low quality reads and remove sequencing adapters. Then, clean reads were aligned to the pig genome defined by NCBI (GCF_000003025.6) with BWA (version 0.7.15-r1140).^55^ The aligned reads were recorded in bam format and processed by samtools (version 1.3.1).^56^ PCR duplicates were removed using sambamba (version 0.6.6).^57^ The software strelka2 (version 2.9.10)^38^ was used to detect somatic mutations in the genome. SNVs and indels were annotated by ANNOVAR (version 2017Jul17).^58^ A Venn diagram was constructed using the R package venn (version 1.11), and a Circos plot of the genomic mutation distribution was constructed using the R package circlize (version 0.4.15).

### Statistical analysis

Data were statistically analyzed by using GraphPad Prism V10.0, and the results were displayed as mean ± SEM or mean ± SD. As for transcriptome sequencing data, more than three replicates were included, and statistical results were presented as mean ± SEM by using R packages. *P*-values were calculated using unpaired *t*-test, and *P* value < 0.05 was considered as statistically significant.

### Supplementary information

Supplementary Materials

## Data Availability

The authors declare that the main data supporting the findings of this study are available within the paper and its Supplementary Information. All sequencing data that support the findings of this study have been uploaded in the NCBI database (accession number: PRJNA816985).
