# Supplementary material for: In vivo evaluation of guide-free Cas9-induced safety risks in a pig model
Source: Signal Transduct Target Ther. 2024 Jul 19;9:184. doi: 10.1038/s41392-024-01905-1 (PMC11258294; doi:10.1038/s41392-024-01905-1)
Supplement: Supplementary file 1 — Supplementary Materials [file 41392_2024_1905_MOESM1_ESM.docx]

Supplementary Materials for

*In vivo* evaluation of guide-free Cas9-induced safety risks in a pig model

Weikai Ge; Shixue Gou; Xiaozhu Zhao; Qin Jin; Zhenpeng Zhuang; Yu Zhao; Yanhui Liang; Zhen Ouyang; Xiaoyi Liu; Fangbing Chen; Hui Shi; Haizhao Yan; Han Wu; Liangxue Lai; Kepin Wang.

Correspondence to: Kepin Wang (wang_kepin@gibh.ac.cn), Liangxue Lai (lai_liangxue@gibh.ac.cn), Han Wu (wu_han@gibh.ac.cn)

**This PDF file includes:**

Figures. S1 to S11

Tables S1 to S2


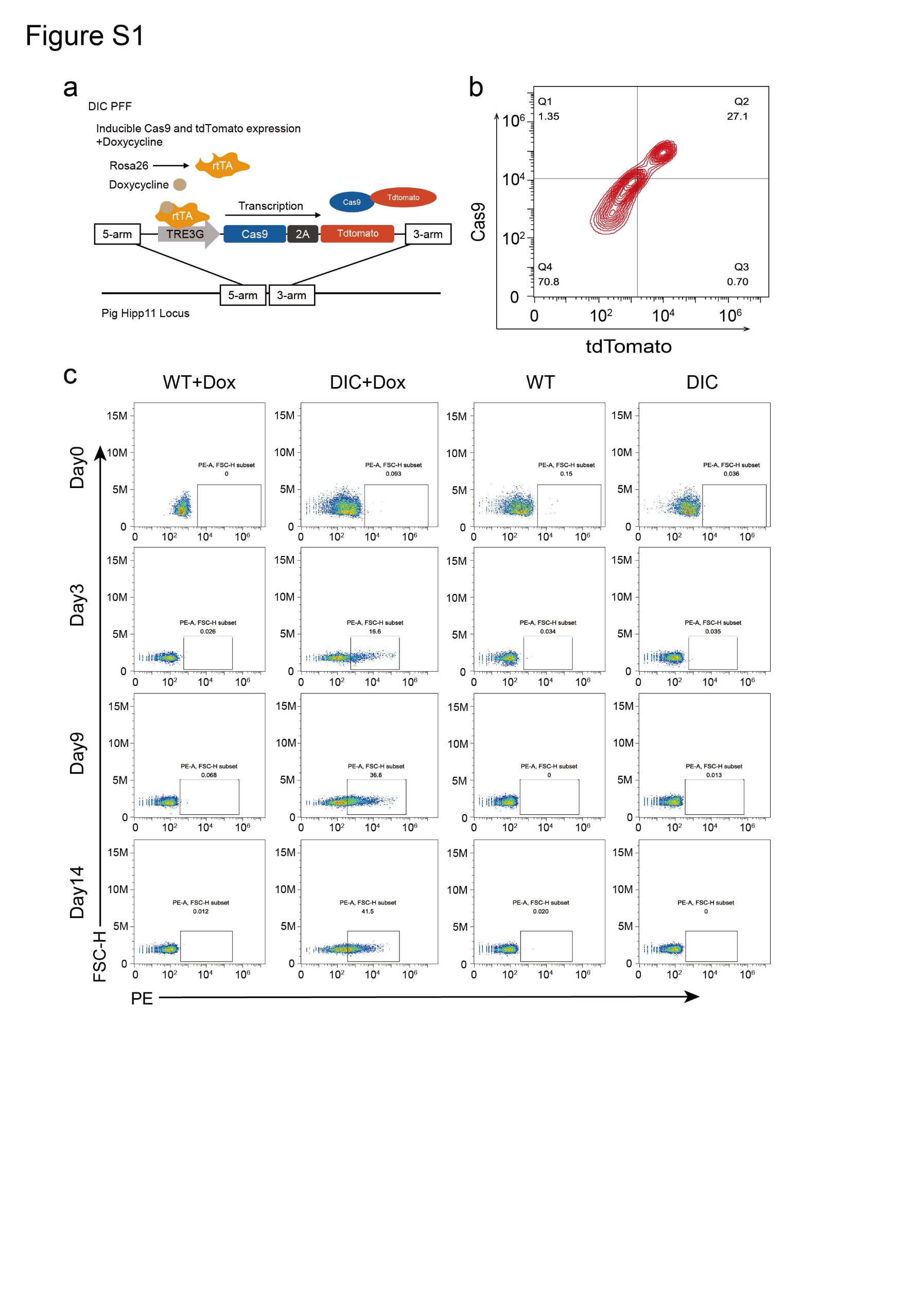


Figure. S1. Analysis of the expression of Cas9 in DIC pigs.

**(a)** Schematic diagram of the expression of Cas9 in DIC pigs. **(b)** Flow cytometry to label the expression of Cas9 and tdTomato in the PBMCs of DIC pigs administered with Dox for 1 week. **(c)** Flow cytometry to detect the ratio of tdTomato-positive cells in the PBMCs of WT and DIC pigs on Day 0, Day 3, Day 9, and Day 14 with or without Dox administration.


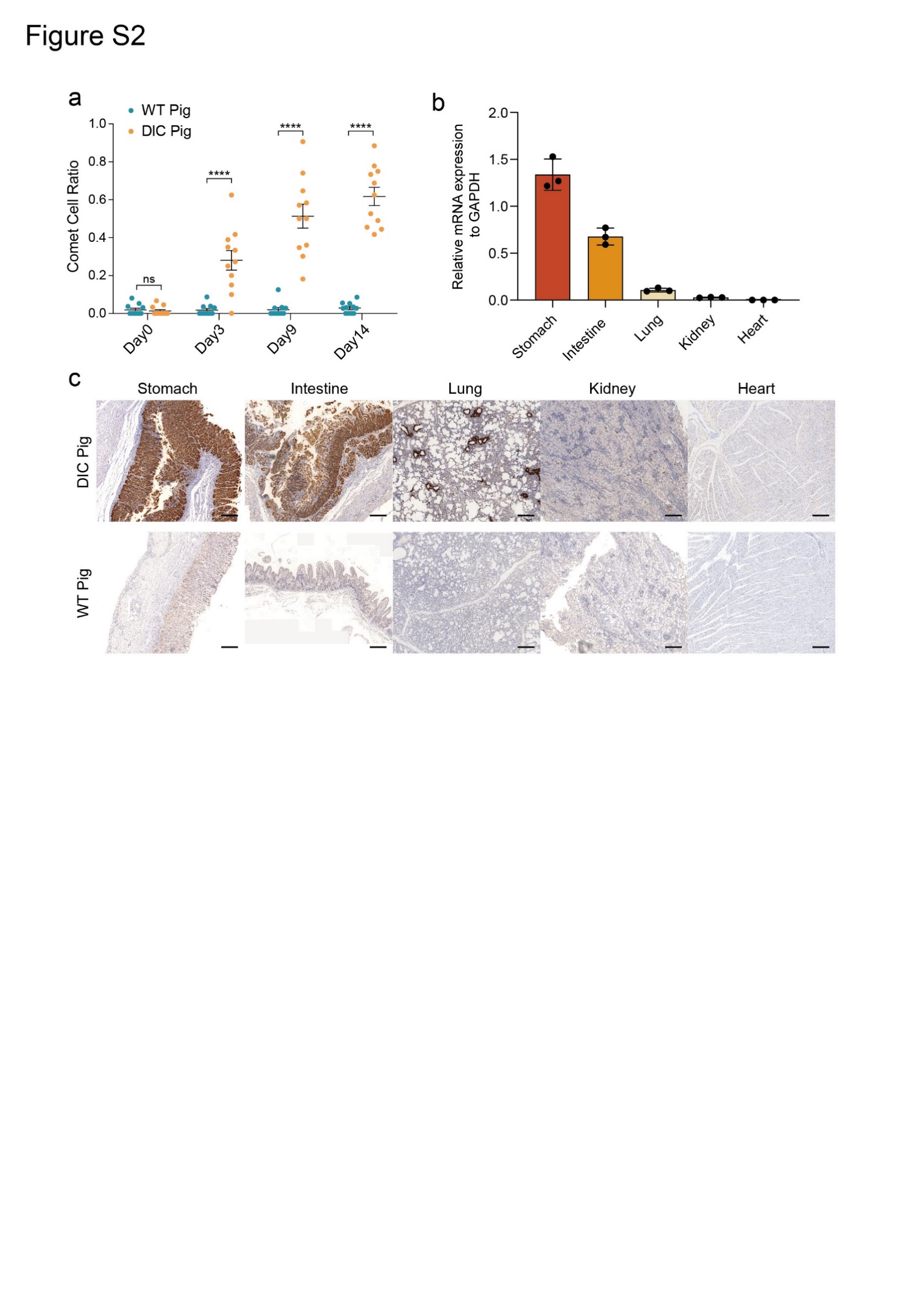
Figure. S2. Analysis of cellular genomic damage in DIC pigs *in vivo*.

**(a)** The ratio of nuclear tailing cells in the PBMCs of WT and DIC pigs on Day 0, Day 3, Day 9, and Day 14 of Dox administration, *****p* < 0.0001, *n* = 11, unpaired *t* test, data represent the means, and error bars correspond to SD. **(b)** Characterization of the Cas9 expression in the stomach, intestine, lungs, kidneys, and heart of DIC pigs by quantitative PCR. **(c)** Characteri-zation of the Cas9 expression in the stomach, intestine, lungs, kidneys, and heart of DIC pigs by IHC staining. Scale bar = 200 μm.


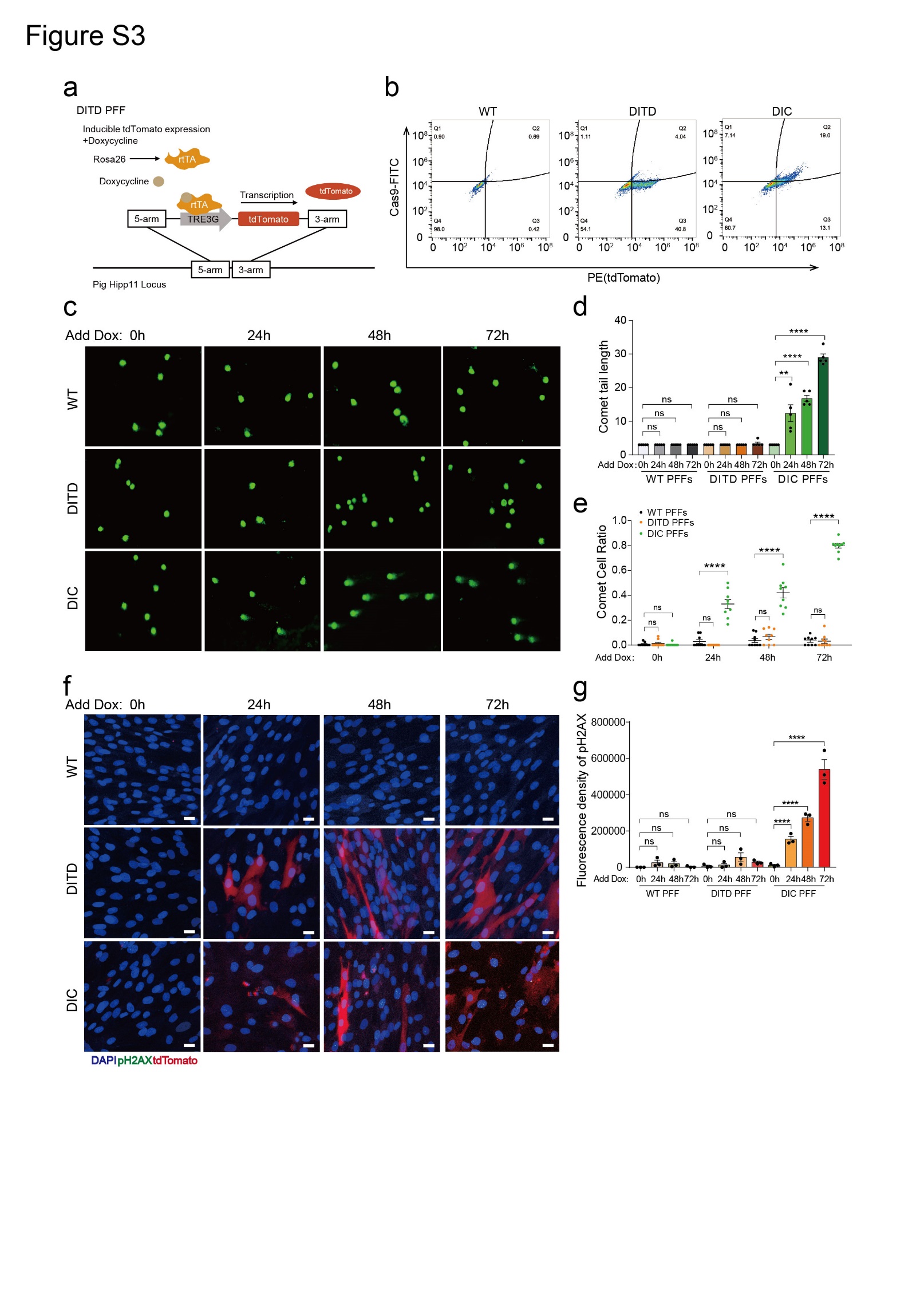
Figure. S3. Analysis of cellular genomic damages in WT, DITD, and DIC PFFs.

**(a)** Schematic diagram for DITD PFFs. **(b)** Flow cytometry to label the expression of Cas9 and tdTomato in WT PFFs, DITD PFFs, and DIC PFFs post 3-day Dox treatment. **(c)** Comet assay detects the WT, DITD, and DIC PFFs at 0 h, 24 h, 48 h, 72 h, and 96 h after Dox treatment. **(d)** Quantification of tail length of WT, DITD, and DIC PFFs at 0 h, 24 h, 48 h, 72 h, and 96 h after Dox treatment, ***p* < 0.0021, *****p* < 0.0001, *n* = 5, unpaired *t* test. **(e)** The ratio of nuclear tailing cells in the PFFs of WT, DITD, and DIC in vitro after 0 h, 24 h, 48 h, and 72 h after Dox administration, *****p* < 0.0001, *n* = 9, unpaired *t* test. **(f)** Fluorescent microscopy images of pH2AX (green), tdTomato (red) and DAPI (blue) in WT, DITD, and DIC PFFs at 0 h, 24 h, 48 h, and 72 h after Dox administration. Scale bar = 20 μm. **(g)** Quantification the fluorescence density of pH2AX in WT, DITD, and DIC PFFs at 0 h, 24 h, 48 h, and 72 h after Dox administration, *****p* < 0.0001, *n* = 3, unpaired *t* test. Data represent the means, and error bars correspond to SEMs.


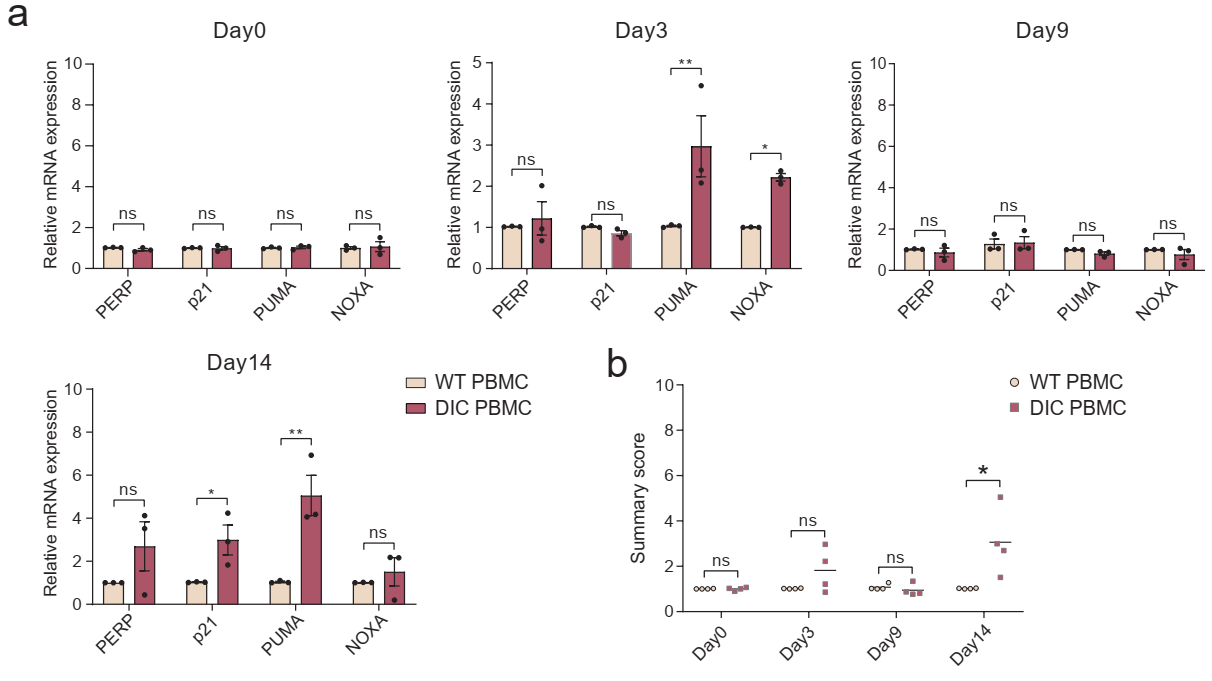
 Figure. S4. Analysis of the expression of P53 target genes in the PBMCs of DIC pigs.

**(a)** Q-PCR analysis of the expression of the P53 target genes *PERP*, *p21*, *PUMA*, and *NOXA* in the PBMCs of WT and DIC pigs at four time points along Dox administration, **p* < 0.0332, ***p* <0.0021, unpaired *t* test. **(b)** The average activation of P53 transcriptional targets in PBMCs, **p* < 0.0332, unpaired *t* test. Data represent the means, and error bars correspond to SEMs.


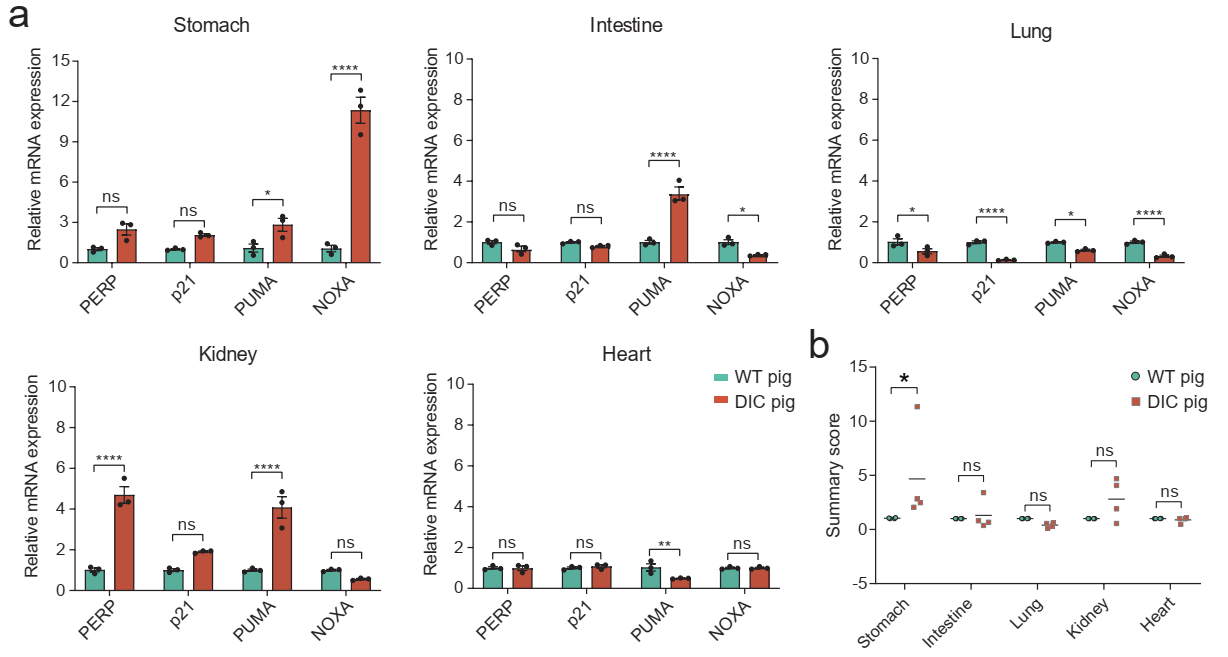
 Figure. S5. Analysis of the expression of P53 target genes in the solid organs of DIC pigs.

**(a)** Q-PCR analysis of the expression of the P53 target genes *PERP*, *p21*, *PUMA*, and *NOXA* in the stomach, intestine, lungs, kidneys, and heart of the WT and DIC pigs after 2 weeks of Dox administration, **p* < 0.0332, **** *p* < 0.0001, unpaired *t* test. **(b)** The average activation of P53 transcriptional targets in each solid organ tissue, **p* < 0.0332, unpaired t test. Data represent the means, and error bars correspond to SEMs.


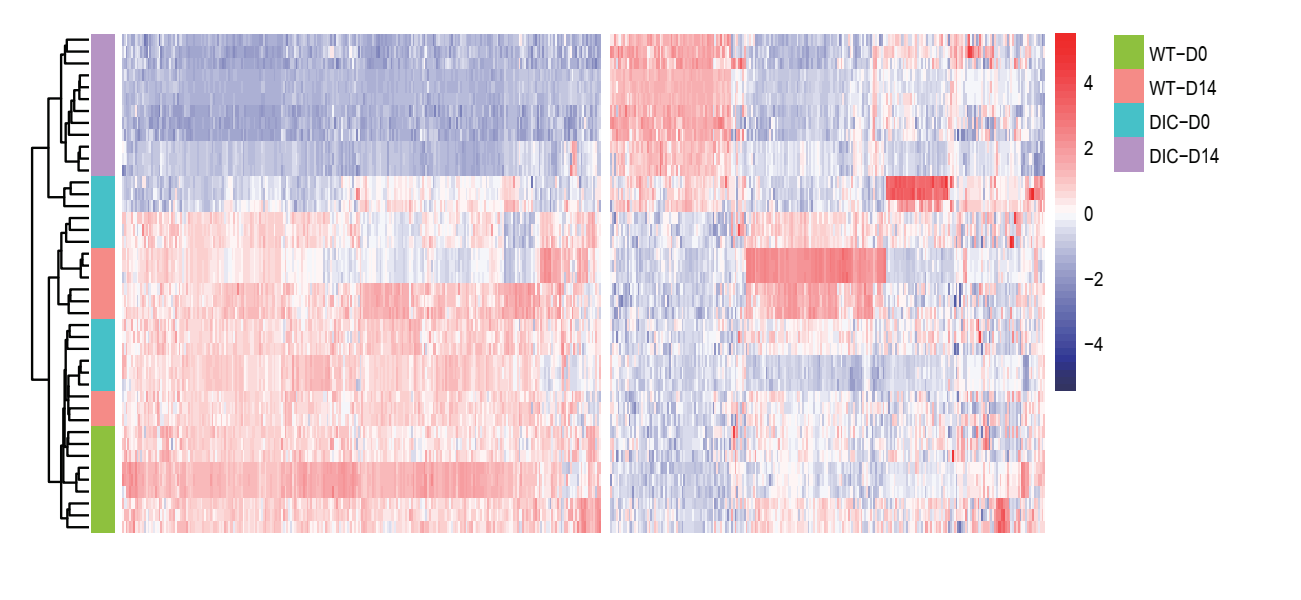
Figure. S6. Hierarchical clustering analysis of RNA-Seq samples from the PBMCs of DIC and WT pigs after administration of Dox for 0 and 14 days.


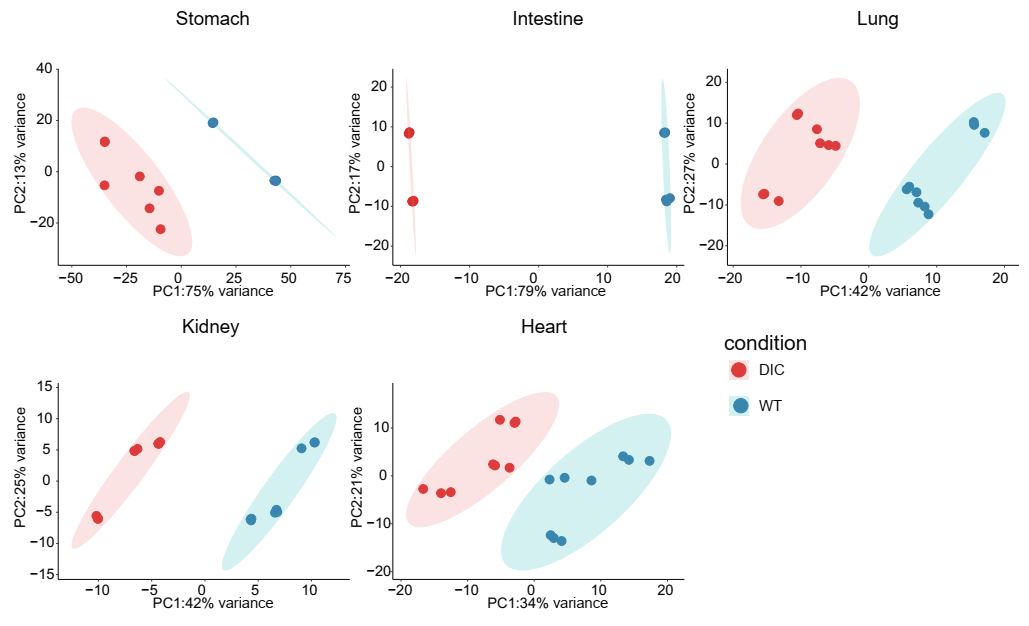
Figure. S7. Principal component analysis of stomach, intestine, lungs, kidneys, and heart samples from DIC and WT pigs after administration of Dox for 14 days.


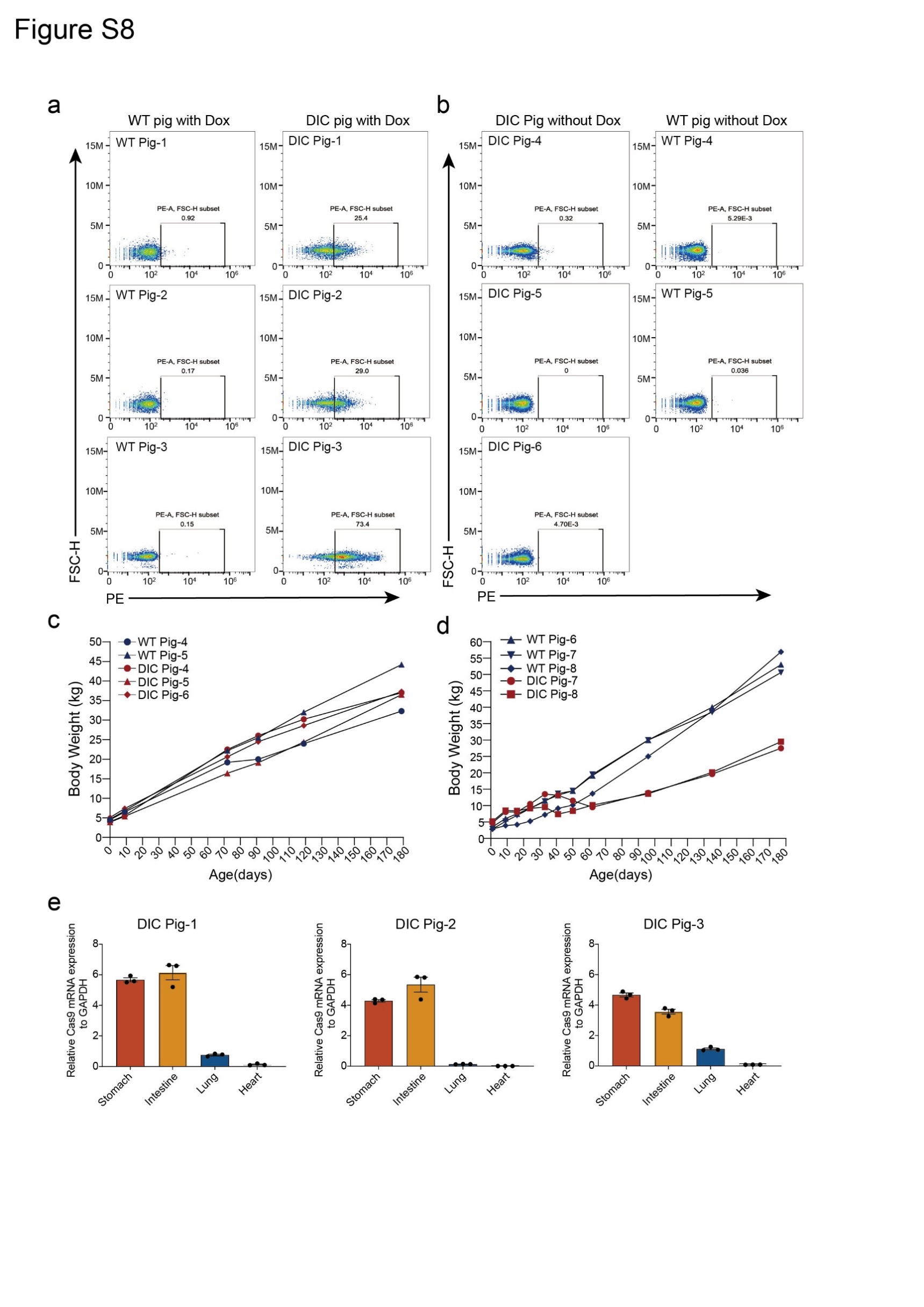


Figure. S8. Analysis of the expression of Cas9 in DIC pigs with long-term Dox administration.

**(a,b)** Flow cytometry to detect the ratio of tdTomato-positive cells in the PBMCs of WT and DIC pigs with or without Dox administration for one month.  **(c)** The growth curves of DIC pigs (DIC Pig-4, DIC Pig-5 and DIC Pig-6) and age-matched WT pigs (WT Pig-4 and WT Pig-5) without Dox feeding. **(d)** The growth curves of DIC pigs (DIC Pig-7 and DIC Pig-8) and age-matched WT pigs (WT Pig-6, WT Pig-7 and WT Pig-8) fed Dox. **(e)** The expression of Cas9 in the stomach, intestine, lung, and heart of DIC Pig-1, DIC Pig-2 and DIC Pig-3. Data represent the means, and error bars correspond to SEMs.


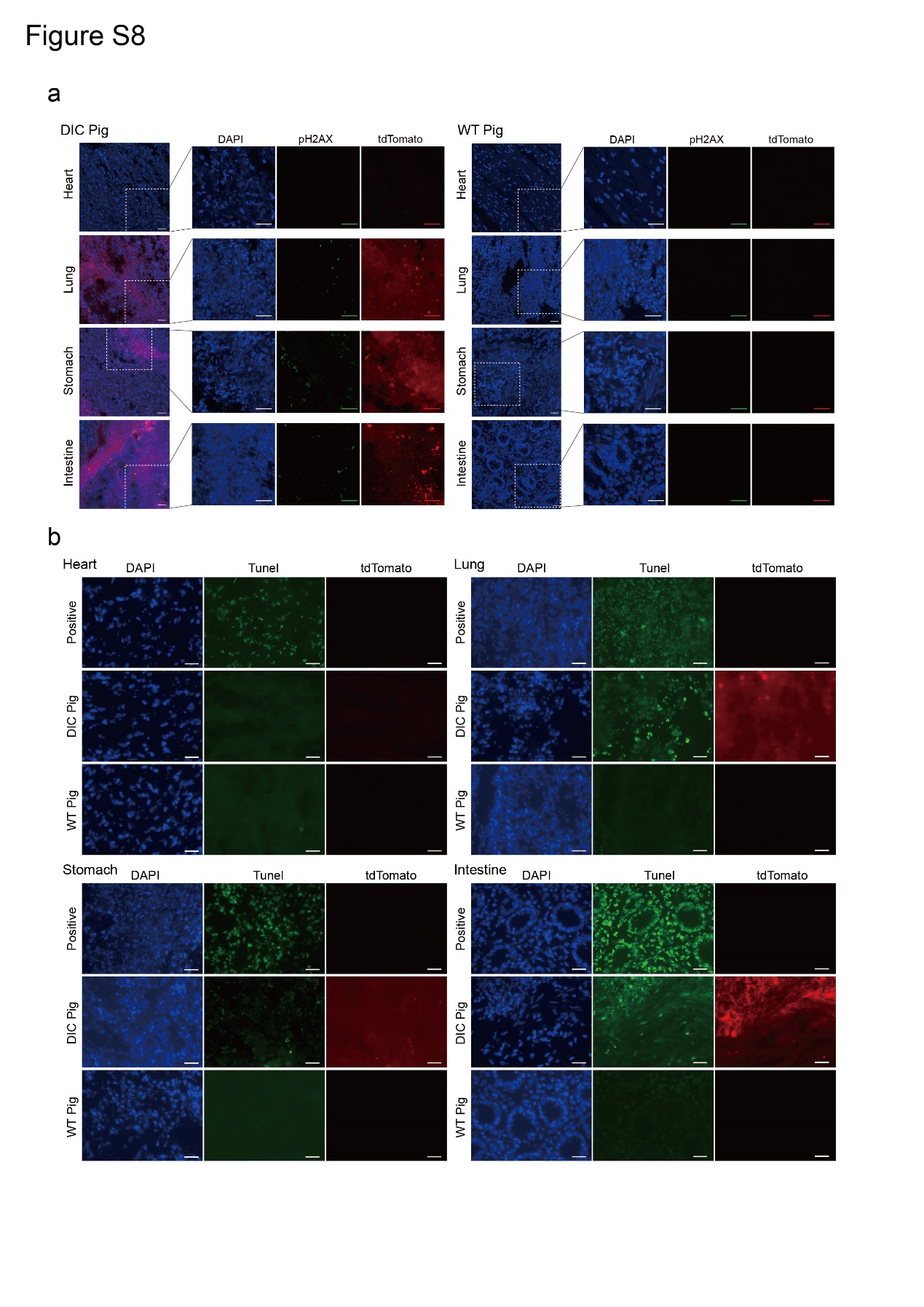
 Figure. S9. Analysis of DNA damage in the solid organs of WT and DIC pigs with long-term Dox administration.

**(a)** Fluorescence microscope images of pH2AX (green), tdTomato (red), and DAPI (blue) in the stomach, intestine, lungs, and heart of WT and DIC pigs with long-term Dox administration. Scale bar = 20 μm.  **(b)** TUNEL assay to detect apoptotic cells in the stomach, intestine, lungs, and heart of WT and DIC pigs with long-term Dox administration. Scale bar = 20 μm.


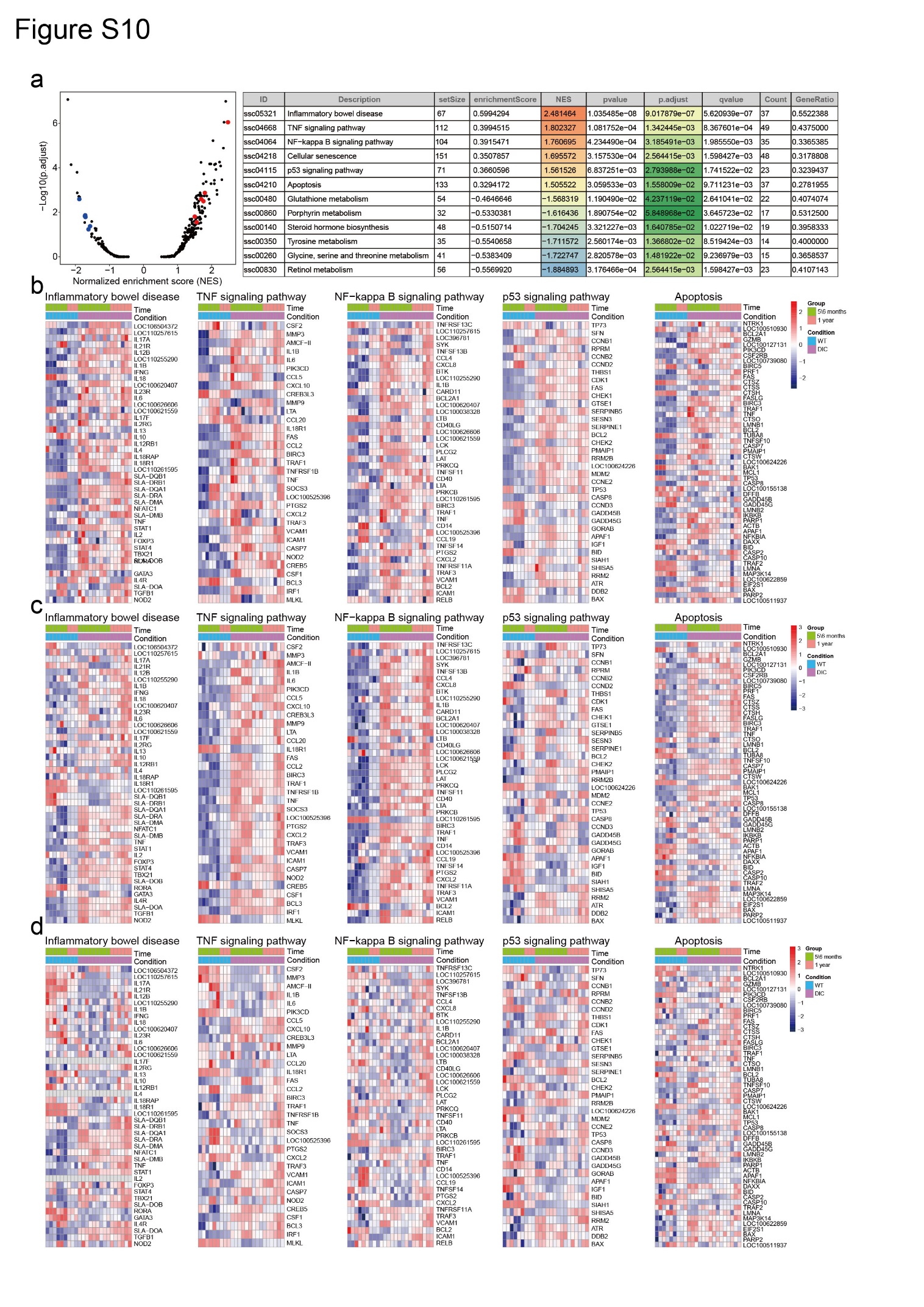

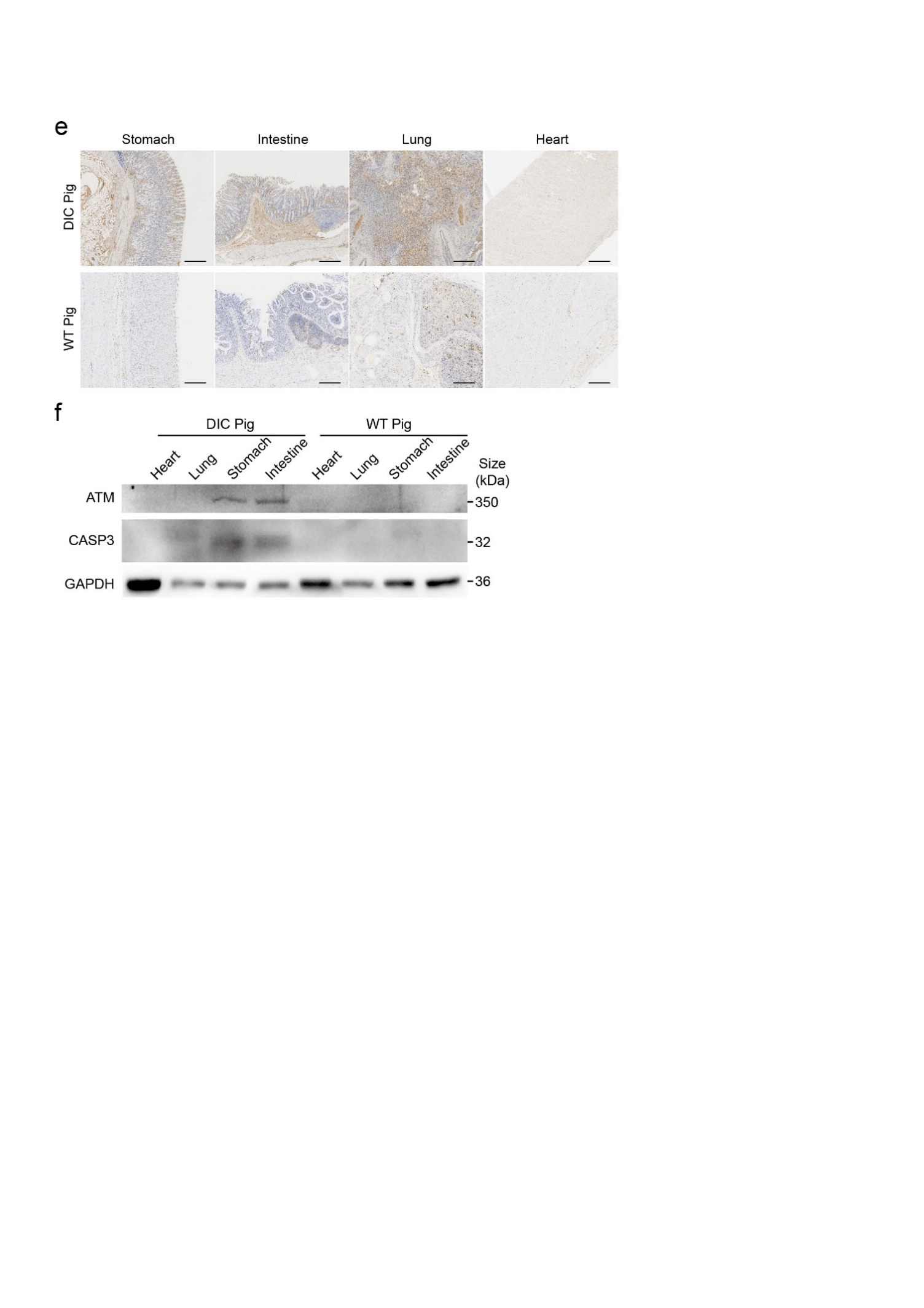
Figure. S10. Transcriptome sequencing analysis of the solid organs of DIC pigs with continuous expression of Cas9.

**(a)** The dot plot (left) and detailed table (right) show the results of GSEA of intestine samples from DIC pigs after Dox feeding. **(b-d)** Heatmap showing the relative expression of genes in DDR-related signaling pathways in the intestine (b), lungs (c), and heart (d). The Z score was calculated to indicate relative expression between samples. **(e)** Characterization of the CD68 expression in the stomach, intestine, lungs, and heart of DIC pigs and WT pigs by IHC staining. Scale bar = 250 μm. **(f)** Western blot analysis of ATM and CASP3 proteins in the stomach, intestine, lung, and heart tissues of DIC pigs and WT pigs.


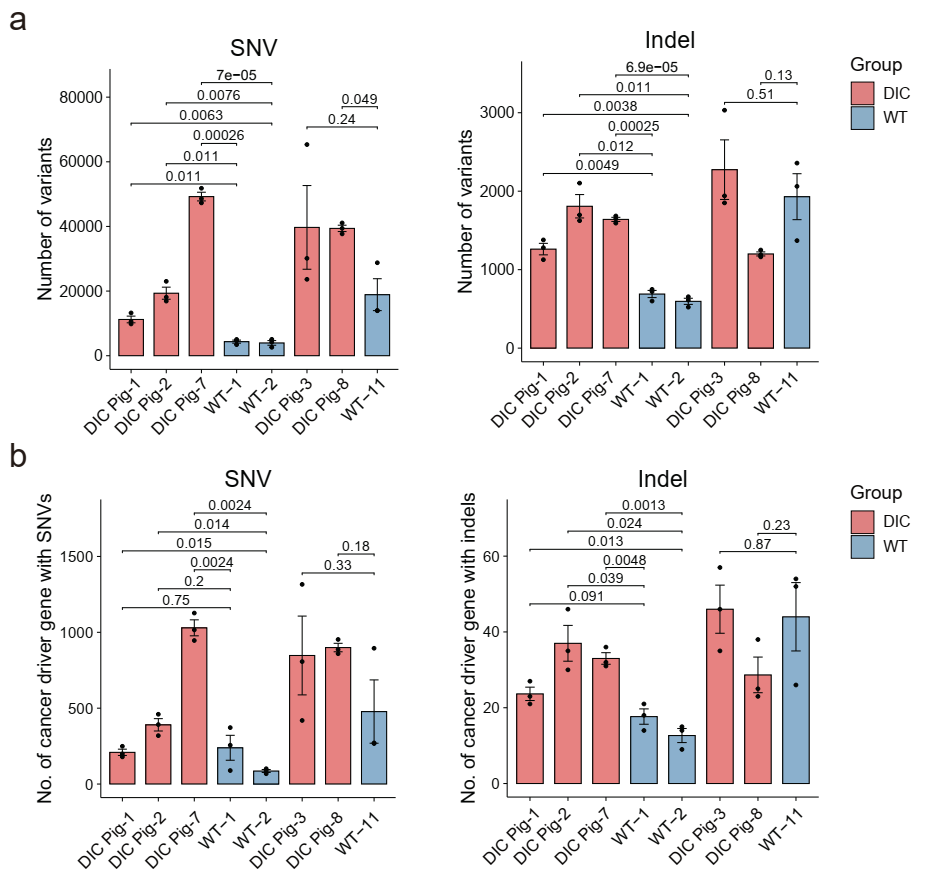
Figure. S11. Genome mutations in DIC pigs with continuous expression of Cas9.

**(a)** The bar plot shows the number of indels and SNVs in stomach samples from the DIC and WT pigs with long-term Cas9 expression. **(b)** The bar plot shows the number of indels and SNVs located on tumor driver genes in stomach samples from the DIC and WT pigs with long-term Cas9 expression. Data represent the means, and error bars correspond to SEMs.

Figure S10f

Table S1. Summary of pigs used in this study.

Table S2. Primers used in this study.
